# Synthesis, Biological Evaluation and Modeling Studies of New Pyrido[3,4-*b*]indole Derivatives as Broad-Spectrum Potent Anticancer Agents

**DOI:** 10.4172/2169-0138.1000143

**Published:** 2017-03-01

**Authors:** Shivaputra A Patil, James K Addo, Hemantkumar Deokar, Shan Sun, Jin Wang, Wei Li, D Parker Suttle, Wei Wang, Ruiwen Zhang, John K Buolamwini

**Affiliations:** 1 Department of Pharmaceutical Sciences, College of Pharmacy, University of Tennessee Health Science Center, 847 Monroe Avenue, Suite 327, Memphis, TN 38163, USA; 2 Department of Pharmaceutical Sciences, College of Pharmacy, Rosalind Franklin University of Medicine and Science, North Chicago, Illinois, 60064, USA; 3 Department of Pharmacology, College of Medicine, University of Tennessee Health Science Center, Memphis, Tennessee, 38163, USA; 4 Department of Biomedical Sciences, School of Pharmacy, Texas Tech University Health Sciences Center, Amarillo, TX 79106, USA; 5 Cancer Biology Center, School of Pharmacy, Texas Tech University Health Sciences Center, Amarillo, TX 79106, USA

**Keywords:** Synthesis, Pyrido[3,4-*b*]indole derivatives, β-carbolines, Structure-activity relationships, Anticancer agents, Colon cancer, Pancreatic cancer, Breast cancer, Prostate cancer, Melanoma, Lung cancer, Cell lines, Molecular docking

## Abstract

**Objective:**

There is an urgent need drugs against particularly difficult to treat solid tumors such as pancreatic, triple negative breast, lung, colon, metastatic prostate cancers and melanoma. Thus, the objective of this study was to synthesize compounds based computational modeling that indicated the pyrido[3,4-*b*]indole class bind to MDM2, a new cancer target for which there are still no drug on the market.

**Methods:**

Compounds were synthesized by established methods and tested for antiproliferative activity against a broad range of human cancer cell lines, comprising HCT116 colon, HPAC, MIA PaCa-2 and Panc-1 pancreatic, MCF-7 and MDA-MB-468 breast, A375 and WM164 melanoma, A549 lung, and LNCaP, DU145 and PC3 prostate cancer lines. Computational docking was also undertaken.

**Results:**

The novel pyrido[3,4-*b*]indoles synthesized exhibited a clear SAR with regards to antiproliferative activity, with potent broad-spectrum anticancer activity with IC_50_s down to 80, 130, 130 and 200 nM for breast, colon, melanoma and pancreatic cancer cells, respectively. 1-Naphthyl at C1 combined with methoxy at C6 provided the best antiproliferative activity. Thus, compound **11** (1-naphthyl-6-methoxy-9*H*-pyrido[3,4-b]indole) showed the highest potency. A mechanistic feature of the compounds as a group is a strongly selective G2/M cell cycle phase arrest. Docking at on MDM2 suggested a hydrogen bond interaction between the 6-methoxy Tyr106, hydrophobic interaction with Val93, pi-pi stacking interactions with Tyr100 and His96 and hydrophobic interactions with Leu54 and Ile99. An N9-methyl group disrupted binding interactions, such as H-bond interactions involving the N9 hydrogen.

**Conclusion:**

We have identified a novel series of pyrido[3,4-*b*]indoles with potent broad spectrum anticancer activity towards the most aggressive and difficult to treat cancers including metastatic pancreatic cancer, non-small cell lung cancer, triple negative breast cancers, and BRAF^V600E^ mutant melanoma, as well as metastatic colon and prostate cancers. There was also evidence of selectivity towards cancer cells relative to normal cells. These compounds will serve as new leads from which novel therapeutics and molecular tools can be developed for a wide variety of cancers.

## Introduction

On a global scale, cancer is a formidable health problem and its incidence is high and it ranks a close second only to heart disease as the top cause of death in America and worldwide. Presently, one quarter of all deaths in the USA are caused by cancer [1,2]. Cancer is the most complex disease with many types and subtypes affecting different body organs. Cancer is mostly, aggressive and lethal and it is predicted that, over the coming years, its incidence rates will grow significantly in line with the increasing global elderly population, and adaptation of lifestyle choices such sun bathing and tanning. The aggressiveness of cancer has also been met with aggressive therapeutic developmental approaches, with significant progress having been made [3]. In the past several years, we have seen dramatic changes in anticancer drug development. Even though, numerous kinase inhibitors have been discovered recently, and several have been successfully developed for treatment of cancer including imitanib (Gleevec), gefitinib (Iressa) and erlotinib (Terceva), still there is strong demand for the discovery of improved anticancer drugs [4–6]. Medicinal chemistry will play a most important role in the discovery and development of novel potent anticancer drugs. In our initial efforts to join the fight against cancer, we applied medicinal chemistry to go after the p53-MDM2 axis for anticancer drug discovery as it has emerged as potential novel for cancer therapeutics development [7].

**Figure 1:**
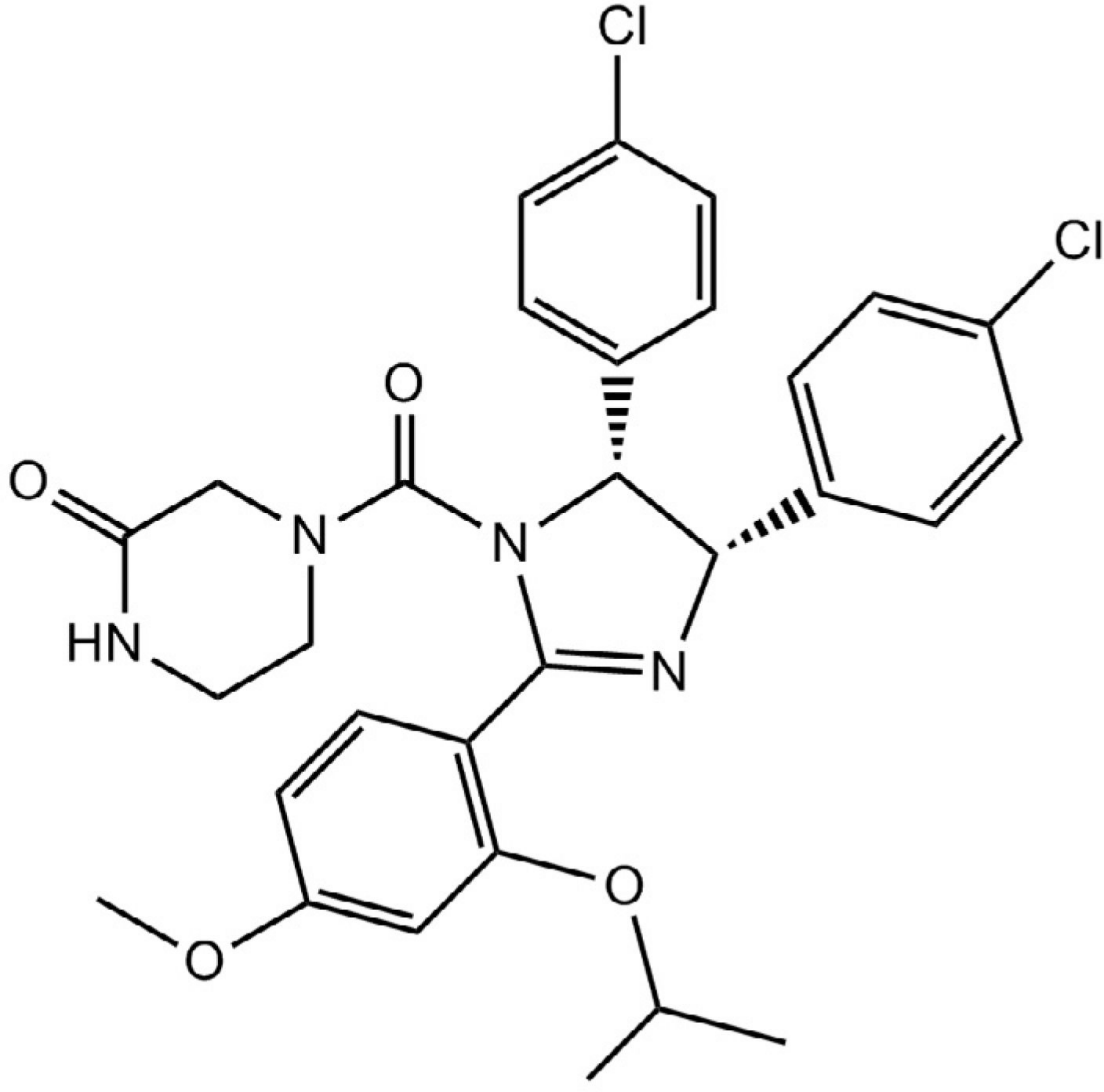
Nutlin3a.

**Figure 2:**
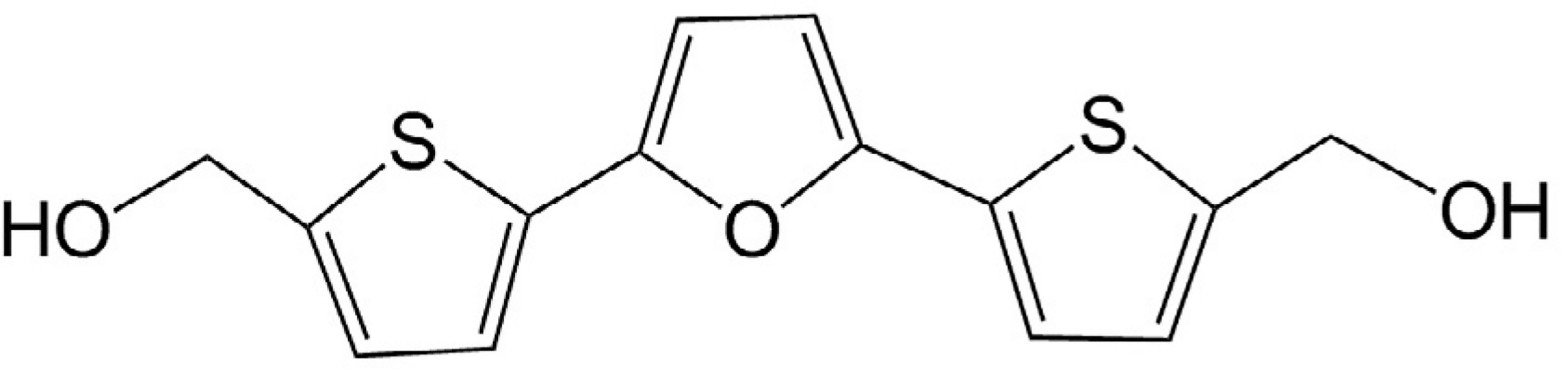
RITA.

The MDM2-p53 pathway offers a promising target for cancer therapy, and although it has been well-validated and clinical trials are being conducted, so far there is no drug on the market that specifically targets the MDM2 pathway(s) [8,9]. Indeed, several targeting strategies have been tried, including blocking MDM2 gene expression, inhibiting MDM2-p53 interaction and modulating MDM2’s E3 ubiquitin ligase activity [10]. Consequently, several MDM2 inhibitors have been identified and shown to have anticancer activity in preclinical settings [11,12]. The majority of MDM2 inhibitors such as nutlin 3a target the MDM2-p53 binding; activating the p53 pathway in cells with wild-type p53 as the major mechanism of action [12]. In addition to its interaction with and regulation of p53, MDM2 is also oncogenic in its own right [13]. Thus, there are a wide variety of ways to search for novel MDM2 inhibitors that disrupt its interaction with p53 and/or inhibit its p53-independent oncogenic functions. Several recent reviews on MDM2 inhibitors have appeared [7,14,15] and although agents acting by differing mechanisms are being pursued such as Nutlin 3a which disrupts p53-MDM2 interaction by binding MDM2 [16]. RITA, which disrupts p53-MDM2 interaction by binding p53 [17] and MEL the field still remains wide open for novel drug discovery, and moreover, no MDM2-targeted drug has yet appeared on the market (Figures 1 and 2).

In our initial attempts, we adopted a structure-based drug design approach to go after this so called “big brother” of p53, the MDM2 protein, the major regulator of the quintessential tumor suppressor p53 [18]. Thus the X-ray 3D atomic coordinates of the crystal structure of the human p53-MDM2 interface complex at 2.3 Å resolution, (PDB ID 1YCR) [19] were used for the initial modeling studies to discover the pyrido[3,4-*b*]indole as a lead class of compounds after structure-based design and follow-up evaluation of compounds for their effects on the expression levels of p53 and p21 proteins as readout using Western blotting (data not shown). The idea being that if the compounds interfered with MDM2’s suppression of p53, then protein levels of p53 and its transcription targets such as p21 waf1 would increase. Indeed, in accordance with our expectations several compounds did that, and the pyrido[3,4-*b*]indole class (β-carbolines) stood out. This encouraging observation gave us the impetus to focus our efforts in the discovery of MDM2 inhibitors on pursuing and development of novel β-carbolines for development of novel targeted anticancer agents. β-Carbolines constitute key structural elements of several naturally occurring indole alkaloids, many of them being of enormous physiological and therapeutic significance. In the past few years several research groups have reported significant antitumor activities of variously substituted β-carbolines [20–25], and the β-carboline nucleus is present as either the fully oxidized form as in harmine and manzamine (I) [26] or in a reduced form as in eudistomine K (II) [27,28] and azatoxin (III) which have displayed cytotoxic activities against various cancer cell lines [29].

## Chemistry and Biology

**Figure 3:**
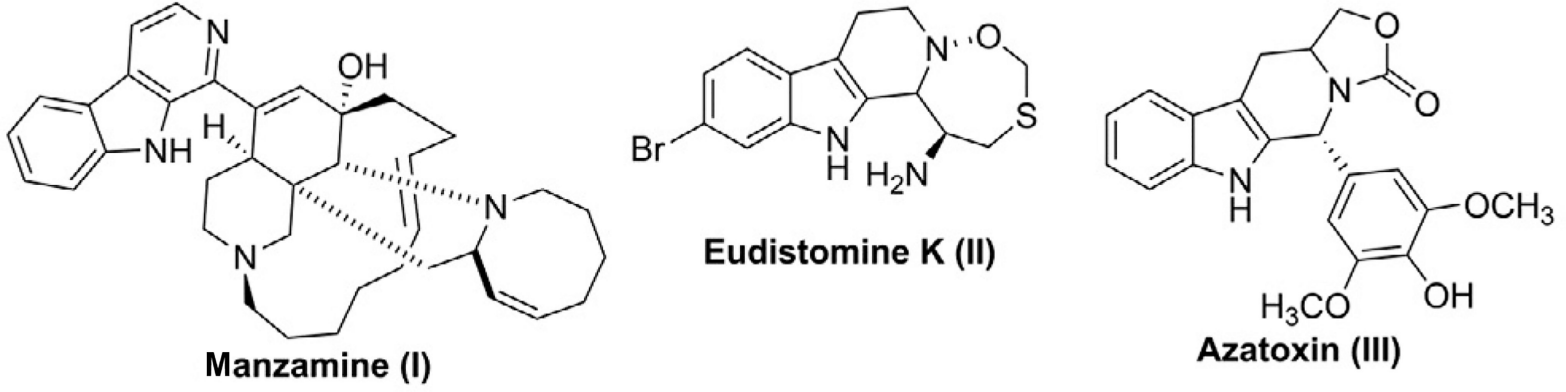
Some β-carboline derivative cytotoxic natural products.

**Scheme 1:**
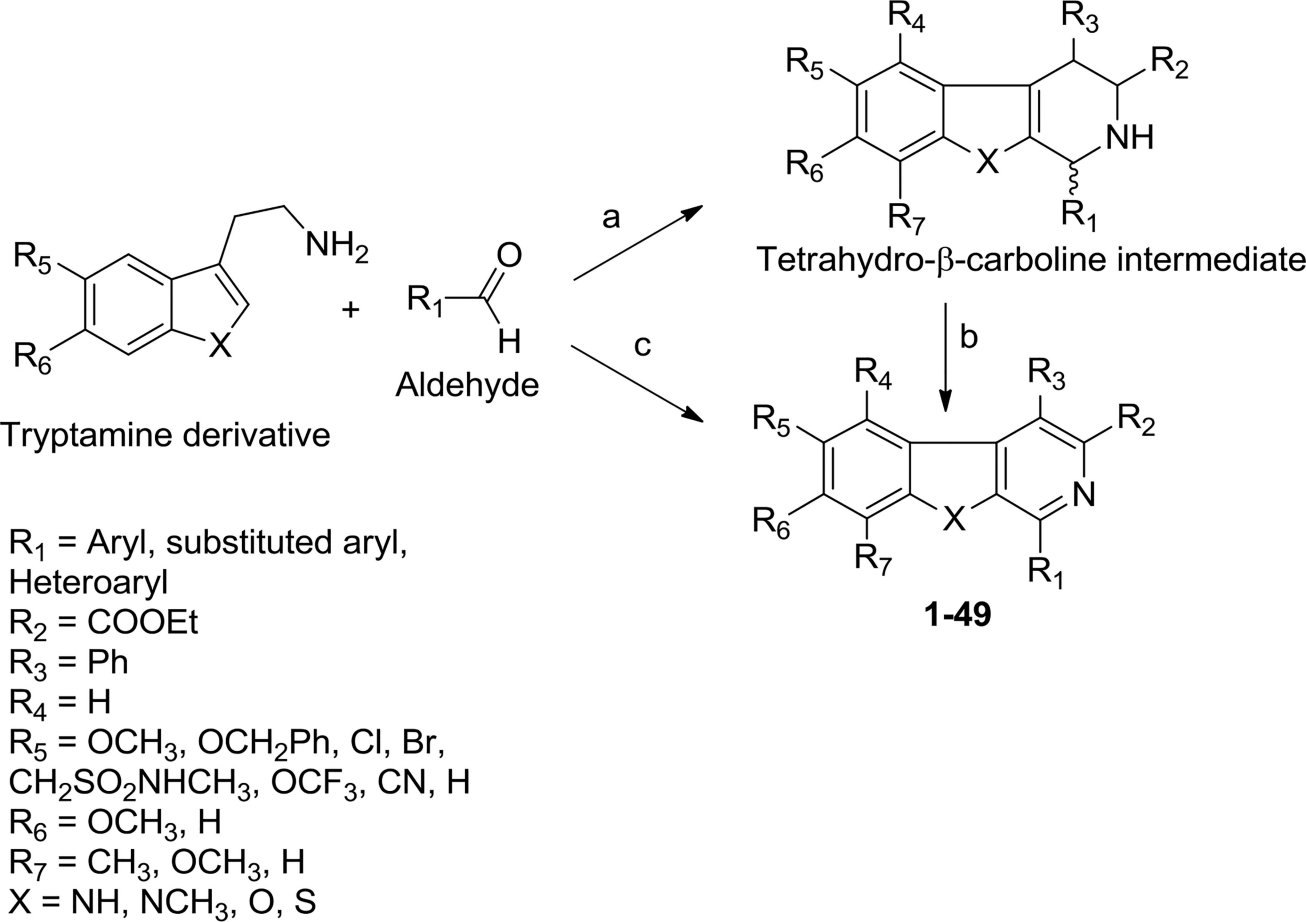
Synthesis of new pyrido[3,4-*b*] indole (β-carboline) derivatives.

We designed and synthesized a series of new β-carbolines bearing various substitutions at the C1, C6 and C7 positions on the pyrido[3,4-*b*]indole core. The selective introduction of various aryl/heteroaryl at these positions was successfully achieved by the reaction of aryl/heteroaryl substituted aldehydes with available substituted trypatmines using Pictet–Spengler reaction followed by oxidation (Scheme 1 a and b); or by using a one-pot microwave assisted coupling conditions (Scheme 1c) (Figure 3).

This method was first utilized with indole bases such as tryptamine to prepare tetrahydro-β- carbolines and since then has become one of the important reactions that have been extensively used for the construction of β-carbolines [30–32]. We condensed the commercially available tryptamines or tryptamine hydrochlorides with aromatic aldehydes in the presence of trifluoroacetic acid in anhydrous tetrahydrofuran to get the tetrahydro-β-carbolines in the first step. The tetrahydro-β-carbolines were oxidized in the presence of Pd/C in xylene at reflux conditions to obtain the desired β-carbolines in 50–55% overall yields. In the alternative synthetic procedure involving microwave-assisted one pot method [33] for the preparation of some of these new β-carbolines, we coupled various substituted tryptamines or tryptamine hydrochlorides with aromatic aldehydes in presence of the bifunctional catalyst (Pd/C/K-10) for the one pot cyclization/dehydrogenation to get β-carbolines in yields of 50–75%. Modification at the C1-position received the most effort, mainly consisting of substitutions with bicyclic aryl or heteroaryl groups such as naphthalene, quinoline, isoquinoline and quinoxaline with the C1 position attachment position on the substituent rings being varied as well [Scheme 1].

## Structure-activity relationships

After, synthesis and characterization, we tested the new series of β-carbolines for anticancer activity to obtain a structure-activity relationship regarding their antiproliferative activity with the expectation that since p53 and its target genes which are important in apoptosis pathways in cancer cells, our compounds will exhibit anticancer effects. The antiproliferative activity of the compounds was thus evaluated in eight cancer cell lines representing four types of human cancer (colon, pancreatic, breast and melanoma). Table 1 shows the IC_50_ values of the compounds tested against the colon and pancreatic cancer cell lines, while Tables 2 and 3 show the antiproliferative IC_50_ values for selected compounds tested against the breast cancer and the melanoma cell lines, respectively. The structural changes produced compounds with a wide ranging antiproliferative activity with IC_50_ values going from 80 nM for the highest antiproliferative activity to greater than 50 µM, giving a fold difference of at least 625. Further, there were also significant chemosensitivity differences among the cancer cells to the compounds, with the HCT116 colon cancer, the WM164 metastatic melanoma and the MDA-MB-468 metastatic triple negative breast cancer cell lines being the most sensitive (Table 1).

The most active compound was 6-methoxy-1-(naphthalen-1-yl)-9*H*-pyrido[3,4-b]indole (11) (R_2_=OCH_3_ and R_1_=1-naphthyl) with IC_50_ values of 0.13, 0.29, 0.20, 0.29, 0.26, 0.08, 0.23 and 0.13 µM towards HCT116, HPAC, MIA PaCa-2, Panc-1, MCF-7, MDA-MB-468, A375 and WM164 cell lines, respectively. It is impressive that all the metastatic pancreatic cancer cell lines were all sensitive to this compound at submicromolar level concentrations. Moreover, it is intriguing that not only that the breast cancer cell lines were very sensitive to compound **11**, but the more aggressive MDA-MB-468 triple negative breast cancer breast cancer cell line was more sensitive than the less aggressive MCF-7 breast cell line (Tables 2 and 3).

Replacement of the C1 naphthyl to a 4-quinolyl substituent produced compound **2**, also with potent antiproliferative activity, exhibiting IC_50_ values of 0.51, 0.56, 0.49 and 0.58 µM for HCT116, HPAC, MIA PaCa-2 and Panc-1, respectively; quite comparable to the lead compound (**11**). Other quinoline analogs such as compounds **1, 9** and **10** displayed moderate activity towards all the cell lines except compound **13**, which demonstrated very good activity for HCT116 (0.90 µM). The isoquinoline substituted compound **7** showed moderate activity towards all the cell lines (HCT116: 5.70; HPAC: 7.37; MIA PaCa-2; 4.70; Panc-1: 10.46 µM) whereas other isoquinoline analogs, **8, 15** and **16** displayed poor activity. This highlights the importance of the ring nitrogen, which has an influence on electron distribution important for receptor binding. The monocyclic analogs compounds **23** and **24** had low activity in both colon and pancreatic cell lines. This implies also that the binding region of the C1 position substituent needs at least bicyclic aromatic or heteroaromatic systems to maintain potent antiproliferative activity.

We also kept the best C1 position aromatic 1-naphthyl substituent in place, and introduced different substituents at the C6 position to explore this area of the compounds. The C6 methoxy substituent (as in compound **11**) was optimal for the series. Replacing it with an indirect electron withdrawing N-methylsulfonamidylmethylene substituent at the C6 position, (compound **34**) showed very good inhibition towards MIA PaCa-2 (0.58 µM) and moderate inhibition towards the other two pancreatic cell lines and the two breast cancer cell lines, but worse than the methoxy substituent. The replacement of the methoxy substituent with the electron withdrawing trifluoromethoxy group (compound **45**), however, drastically reduced antiproliferative activity with no submicromolar inhibitory activity against any of the cancer cell lines. This was also the case in replacing the C6 methoxy group with the electronegative CN substituent (see compound **46**). Interestingly, it does not appear to be a simple electron donating or electron withdrawing effect as the substitution with the electron donating methyl (CH_3_) group led to a sharp drop in antiproliferative activity (see compound **44**).

The removal of methoxy group from A-ring (compound **43**) surprisingly retained moderate activity towards all the pancreatic cell lines (HPAC: 2.47; MIA PaCa-2; 1.70; Panc-1: 9.83 µM) but much lower activity than the methoxy group, suggesting the importance of the methoxy group at C6 position for enhancing activity but not being essential. Moving the methoxy group from the C6 position to the C7 or C8 position decreased anticancer activity, with the C7 position being better than the C8 position (compare compounds **11, 12** and **31**).

Methylation of the N9 nitrogen as in compounds **48** and **49** drastically reduced antiproliferative activity. This shows clearly that the β-carboline 9-position NH is important for potent activity; and might be involved in hydrogen bonding at the binding site. This was suggested by the molecular docking study. In this regard, our compounds are unique in that compared to a series of β-carbolines compounds reported by Cao et al. introduction of an alkyl such as Me or benzyl group on the 9-position nitrogen led to an increase in anticancer activity [34,35]. This suggests that at least for our series a different major mechanism of antiproliferative activity must be operating relative to the series reported by Cao et al. The modeling study we conducted also suggests a detrimental effect of the N-methyl substitution. The other SAR feature that stood out is that replacing the β-carboline N9 by oxygen or sulfur reduced activity significantly. This might also be due to the loss of H-bond donor interaction by NH group at this position. The modeling study we conducted also suggests a detrimental effect of the N-methyl substitution.

The other SAR feature that stood out is that replacing the β-carboline N9 by oxygen or sulfur reduced activity significantly. This might be due to the loss of H-bond donor at this position. The lower activity of the compound **42** relative to **41** supports this notion, as the replacement of an oxygen atom with a sulfur atom increases lipophilicity and would have been expected to increase antiproliferative activity if hydrophobicity alone was the driving force for activity (compare compounds **2** and **11**). It could also be the result of the reduction in electronic complementarity or a combination of these and other factors not apparent at the moment.

The replacement of the methoxy substituent with the electron withdrawing trifluoromethoxy group (compound **45**), however, drastically reduced antiproliferative activity with no submicromolar activity towards any of the cell lines. This was also the case in replacing the C6 methoxy group with the electronegative CN substituent (see compound **46)**. Having a less strong electron withdrawing N-methylsulfonamidylmethylene substituent at the C6 position (compound **32**) showed very good inhibition towards MIA PaCa-2 (0.58 µM) and moderate inhibition towards the other two pancreatic cell lines as well as the breast cancer cell lines, but still worse than the methoxy substituent. Interestingly, it does not appear to be a simple electron donating or electron withdrawing effect as the replacement of the methoxy with the electron donating methyl (CH_3_) group led to a drop in antiproliferative activity (see compound **38**). The removal of the methoxy group from the A-ring (compound **43**) surprisingly retained moderate activity towards all pancreatic cell lines (HPAC: 2.47; MIA PaCa-2; 1.70; Panc-1: 9.83 µM) suggesting that the presence of the methoxy group at C6 position is needed for high potency (activity enhancing) but is not essential for activity. Further, replacement of the C6 methoxy group with the more bulky benzyloxy group (compound **28**) reduced activity only slightly compared to compound **11**, which possibly implies that there is no undue significant steric hindrance by replacing the smaller methoxy group with the benzyloxy group; and suggesting that large groups can be explored at the C6 position. Placing a carbethoxy group (CO_2_Et) at the C3 position led to a drastic reduction in activity (compare compounds **11** and **47**). Placing a phenyl substituent at the C4 position also led to a reduction in activity but only to half the extent to which the carbethoxy substituent at the C3 position reduced the potency (compare compounds **33** and **47**).

HCT116, WM164 and MDA-MB-468 cancer cell lines were the most sensitive to this compound. It is also impressive that aggressive pancreatic cancer cell lines were sensitive at the submicromolar level. It is intriguing that the more aggressive breast cancer cells are more sensitive to this compound than even the less aggressive breast cancer cell line (compare MDA-MB468 and MCF-7 cell lines in Table 2).

Changing R_1_ at C1 position naphthyl attachment to the 2-naphthyl (compound **17**) reduced the activity for all four cell lines, however it still afforded good inhibition towards the HCT116 (0.67 µM) and MIA PaCa-2 (1.36 µM) cell lines. Adding electron donating groups such as methyl and methoxy groups to the naphthyl moiety of compound **11** led to all three compounds (compounds **25–27**) retaining single digit micromolar activity against HCT116 cells with IC_50_ values of 1.80, 2.43 and 2.22 µM for compounds **25, 27** and **26**, respectively.

Compound **25** also exhibited substantial activity against the pancreatic cell line MIA PaCa-2 with an IC_50_ of 2.23 µM. When the naphthyl moiety at the C1 position was changed to an anthracenyl or a phenanthryl moiety (compounds **19** to **22**). All four compounds showed good inhibitory activity towards HCT116, HPAC and MIA PaCa-2 cell lines. Among them, compound **21** showed the best antiproliferative activity towards HCT116, HPAC and MIA PaCa-2 with IC_50_ values of 0.53, 0.54 and 0.84 µM, respectively; which is quite comparable to compound **11**, our lead candidate. Thus, just as in the case of the bicyclic substituents, the angle of the tricyclic substituents also matters which indices specific shape requirements at the C1 position aryl substituent. Thus it is not just a matter of increasing the hydrophobicity but proper positioning of the substituent. Substituting a phenyl ring at the C1 position (compounds **23** and **24**) drastically decreased the activity to nearly 30 to 45 µM range for HCT116, HPAC and MIA PaCa-2 cell lines; and both compounds exhibited IC_50_ values greater than 50 µM for Panc-1 cells. The 4-phenylpyridine biaryl analog compound 30 also showed low inhibitory activity against all pancreatic cancer cell lines, but had good activity against HCT116 cells (IC_50_ 3.0 µM). Substitution at the C8 position is detrimental to activity as both a methoxy and a methyl substituent reduced activity compared to the 6- or 7-position methoxy substituted compounds. With the C6 position methoxy substitution held constant, modifications on the C1 naphthyl group led generally to reduction in antiproliferative activity. For example, introduction of a methyl group at the naphthyl-2-position as seen in compounds **25** and **26**, led to about 10- to 100-fold decrease in activity (compare compound **11** to compound **25**). Similarly, introduction of a methoxy group at the napththyl-6-position as in compound **27** also led to a similar decrease in activity. On the other hand introduction of halogens, fluorine or bromine at the napththyl-5- position as in compounds **36** and **37** had a much less detrimental effect on activity. In fact, in MIA PaCa-2 cells, the difference is very small, with the IC_50_ values for compound **11** and compound **36** being 0.2 µM and 0.33 µM, respectively. These results, suggest several potential reasons to do with steric and electrostatic effects, as well as electron donation and electron withdrawal as well as hydrophobicity effects. Placing an ethyl bridge across the naphthyl-4- and -5-positions and thus creating an acenaphthyl substituent as in compound **39**, also resulted to a reduction in activity.

Among selected compounds tested against breast cancer cell lines, compound **11** again displayed the best antiproliferative activity against both cell lines with IC_50_ values of 0.26 and 0.08 µM for MDA-MB-468 (TNBC) and MCF-7 (estrogen receptor (ER) positive, p53+) cell lines, respectively, with the more aggressive TNBC cell line was more sensitive than the less aggressive ER^+^ cell line. This is counter intuitive and suggests that the compounds probably might be targeting a TNBC driven molecular target. Also interesting, is that compound **43** which is a compound **11** analog lacking the C6 methoxy substituent exhibited significant activity against the breast cancer cell lines (MDA-MB-468: IC_50_ 0.80 µM; MCF7: IC_50_ 1.42 µM). This reinforces the notion that although the C6 methoxy substituent is important for enhancing activity it is not essential. Interestingly, compound **45**, which has an electron withdrawing trifluoromethoxy group in place of the methoxy group of compound **11**, also demonstrated improved activity against the breast cancer cell lines (IC_50_ values of 2.04 µM and 1.11 µM for MCF7 and MDA-MB-468 cells, respectively) relative to the pancreatic cancer cell lines. Here also, N-methylation was detrimental to antiproliferative activity as compound **49** was much less active towards both breast cancer cell lines than its NH counterpart compound **43**, again suggesting the importance of the free NH functionality. In fact, the breast cancer cell lines were more sensitive to the compounds than the colon and pancreatic cancer cell lines, in particular the more aggressive p53 mutant MDA-MB-468 cell line.

Further, we also evaluated the antiproliferative effects of selected compounds against human melanoma skin cancer cell lines A375 and WM164, which are among the most widely used melanoma cell lines with aggressive metastatic behavior [36–38]. These cancer cell lines are both BRAFV600E mutant cells with constitutively activated mitogen-activated protein kinase (MAPK) signaling. BRAFV600E is the most common (~60%) BRAF mutation in human carcinogenesis and accounts for most clinical metastasis [39–41]. The results presented in Table 4, show a potent submicromolar level inhibition of these melanoma cells by some of the compounds, and the SAR trend is generally similar to what was observed in the other cell lines; compound **11** again being the most potent. However, it is interesting that in these melanoma cell lines, the N-methylated compound, **48** exhibited single digit µM IC_50_ values, and did better than it did against the pancreatic and breast cancer cell lines. This implies that apart from compound **11**, which is the most potent compound across the board, depending on the type of cancer, some compounds may be more applicable than others (Table 4).

## Molecular modeling

**Figure 4:**
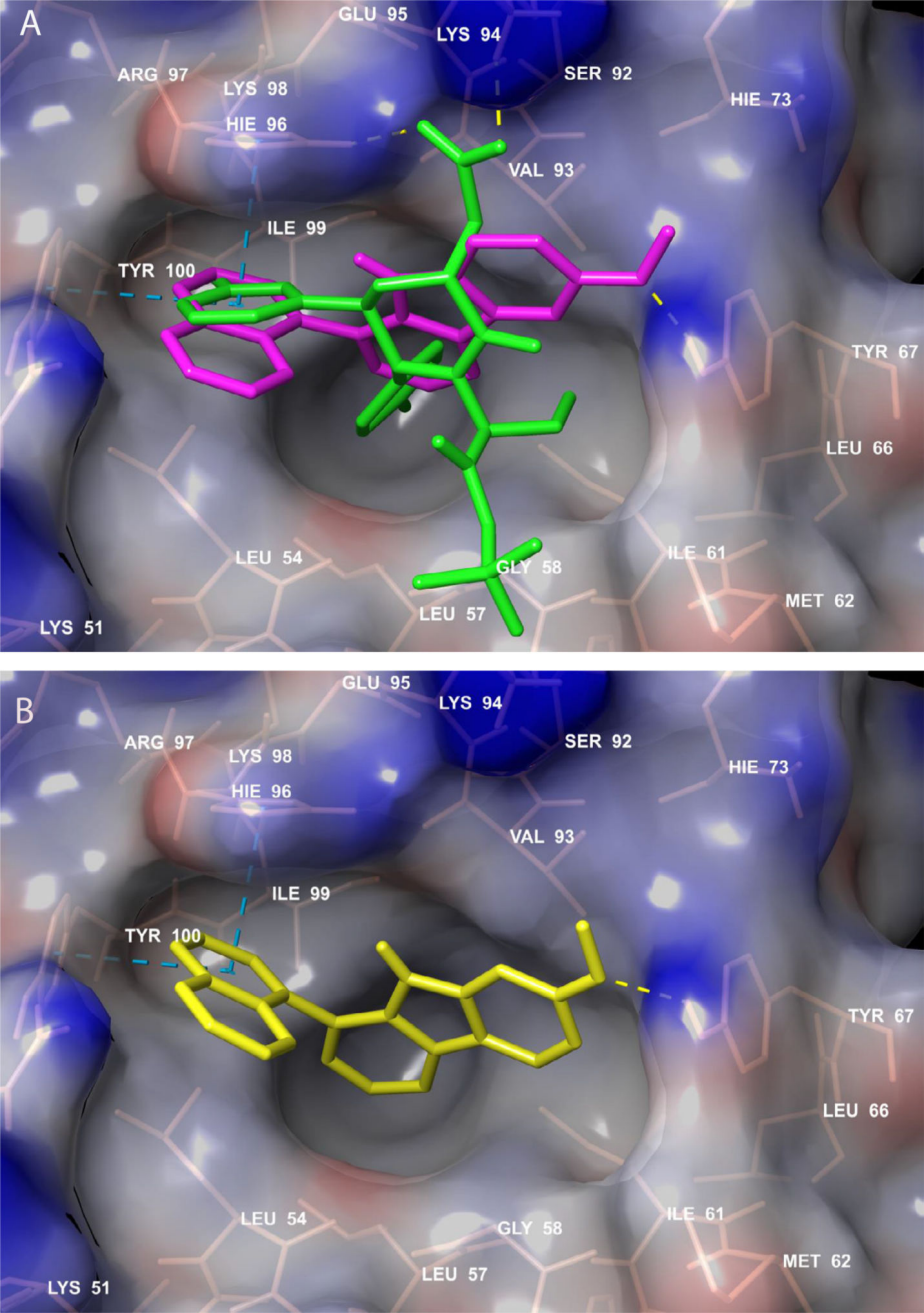

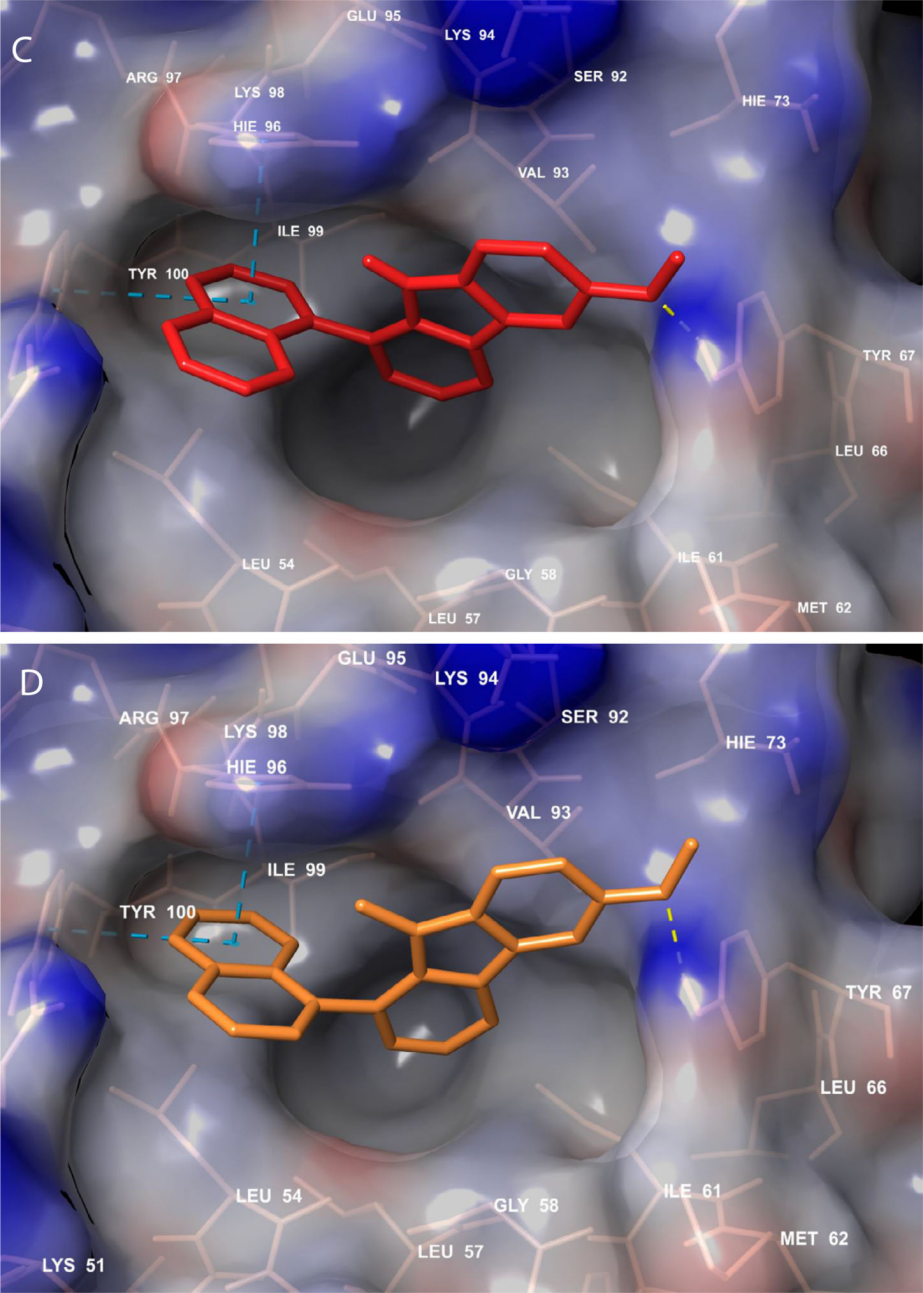
Docking poses of a known MDM2 inhibitor oxopiperidine derivative and representative compounds. (A) Pose of of compound 11 (magenta) superimposed on the docking pose of oxopiperidine derivative (green), in the p53 binding site on MDM2; (B) Docking pose of compound 2 (yellow); (C) docking pose of compound 12 (red); and (D) docking pose of compound 49 (orange). The protein surface was mapped to electrostatic potential using a red- white-blue color scheme (−0.3: minimum (red) and +0.3: maximum (blue). Active site residues interacting with the compounds are shown in pink stick rendering. Pi-pi interactions are shown by cyan dashed lines, while hydrogen bonding between the compounds and the protein is indicated by yellow dashed lines.

To gain further insights into the structural foundations of the SAR, we docked molecules with prominent structural differences from different activity levels for: 1) the most active compounds **1** and **11**; 2) moderately active compound **12** and **3**) low activity compound **49**. The docking poses are shown in Figure 4, while the interaction energies are shown in Table 4.

The most active compound, **11**, docked tightly and made favorable interactions important amino acid residues in the Nutlin 3a binding site on MDM2, resulting in interaction energy of −10.67 Kcal/mol. In this binding pose, the C6 methoxy group interacted with MDM2 Tyr67 through a hydrogen bond (H-bond) acceptor involving the oxygen. The 9-position NH was positioned very close to His 96 at a distance of 2.86 Å, indicating the strong possibility of H- bond donor interaction. The C1 position naphthyl group was engaged in aromatic pi-pi stacking interactions between the ring two active site amino acids residues, Tyr 100 and His 96; and Van der Waals interactions were observed between compound **11** and Gln 24, Leu 54, Gly 58, Ile 61, Tyr 67, Val 93, His 96, Ile 99, and Tyr 100. For compound **12**, which only differs from compound **11** by having the methoxy substituent moved to the 7-position, the docking pose was relatively similar to the pose of compound **11**, with a total binding energy of −9.86 Kcal/mol. The decrease in binding energy for compound **12** may be explained at least in part by the increase in distance (2.10 Å) of the methoxy oxygen from OH group of Tyr 67 which implies a weaker H- bonding of compound **12** with Tyr 67. With compound **11**, this distance is 1.74 Å. The differences are reflected in the H-bond energy for compound **11** being −0.69 kcal/mol, compared to −0.32 kcal/mol for compound 12, a decrease in interaction energy of 0.35 kcal/mol. This hydrogen bonding strength differences might also explain why compounds with a C6 methoxy substituent are generally more potent than their counterparts with a C6 methoxy substituent (compare compounds **2** and **1; 7** and **8; 4** and **5; 11** and **12; 13** and **14; 15** and **16; 17** and **18; 21** and **22**; and **25** and **26**; see Table 1).

With regard to the lower activity of compound **2** relative to compound **11**, that might possibly due to the naphthalene ring in compound **11** being more hydrophobic than the quinoline ring of compound **2**, as they are both predicted to bind in the hydrophobic pocket of MDM2 occupied by the chlorophenyl rings of Nutlin 3a in the MDM2-Nutlin 3a complex crystal structure. In addition, not only does the introduction of a nitrogen atom into the ring system decrease activity, but the position of the nitrogen in the ring also matters. Thus, whereas the 4-quinolyl system in compound **2** still maintained relatively high potency in comparison with compound **11**, the, 3- and 5-quinolyl compounds, **7** and **13**, respectively, exhibited substantially lower activities.

Introduction of two nitrogen atoms into the bicyclic ring system as in compounds **4** and **5**, produced compounds with very low activities. This is likely due to the decrease in hydrophobicity with the introduction of more nitrogen atoms that decrease the hydrophobic interactions. Placing a methyl group on the N9 nitrogen of compound **11**, sharply decreased antiproliferative activity. This is reflected in the docking energies showing a decrease in binding of the N-methyl derivative of **11**, compound **49**, with the binding energy going from −10.48 Kcal/mol for compound **11** to −8.82 Kcal/mol for compound **49** (Table 4). This could be due to steric clashing effects of the N-methyl group as well as a decrease in the pi-pi interactions of the 1-position naphthalene ring with His 96 (HIE 96) and Tyr 100 as the distances between it and these residues increase.

With the observed SAR, we conclude that in this series of β-carbolines, having the methoxy at C6 position provides superior anticancer activity compared to C7 or C8 methoxy analogs, indicating the importance of the position of the methoxy functionality for exerting the antiproliferative activity. Secondly, the naphthyl group at C1 position is very important to retaining high potency as introduction of nitrogen-containing heteroaryl groups exerted lower antiproliferative activity. This study has yielded compound **11** (which is designated SP141) as a candidate that we are further developing in preclinical experimental therapeutic models for pancreatic cancer, and breast cancer [41,42].

Certainly there is still room for SAR development, pharmacologic and mechanistic studies of this particular class of substituted pyrido[3,4-*b*]indole derivatives which we are currently pursuing.

## Cancer cell cycle perturbation and cancer cell selectivity

**Figure 5:**
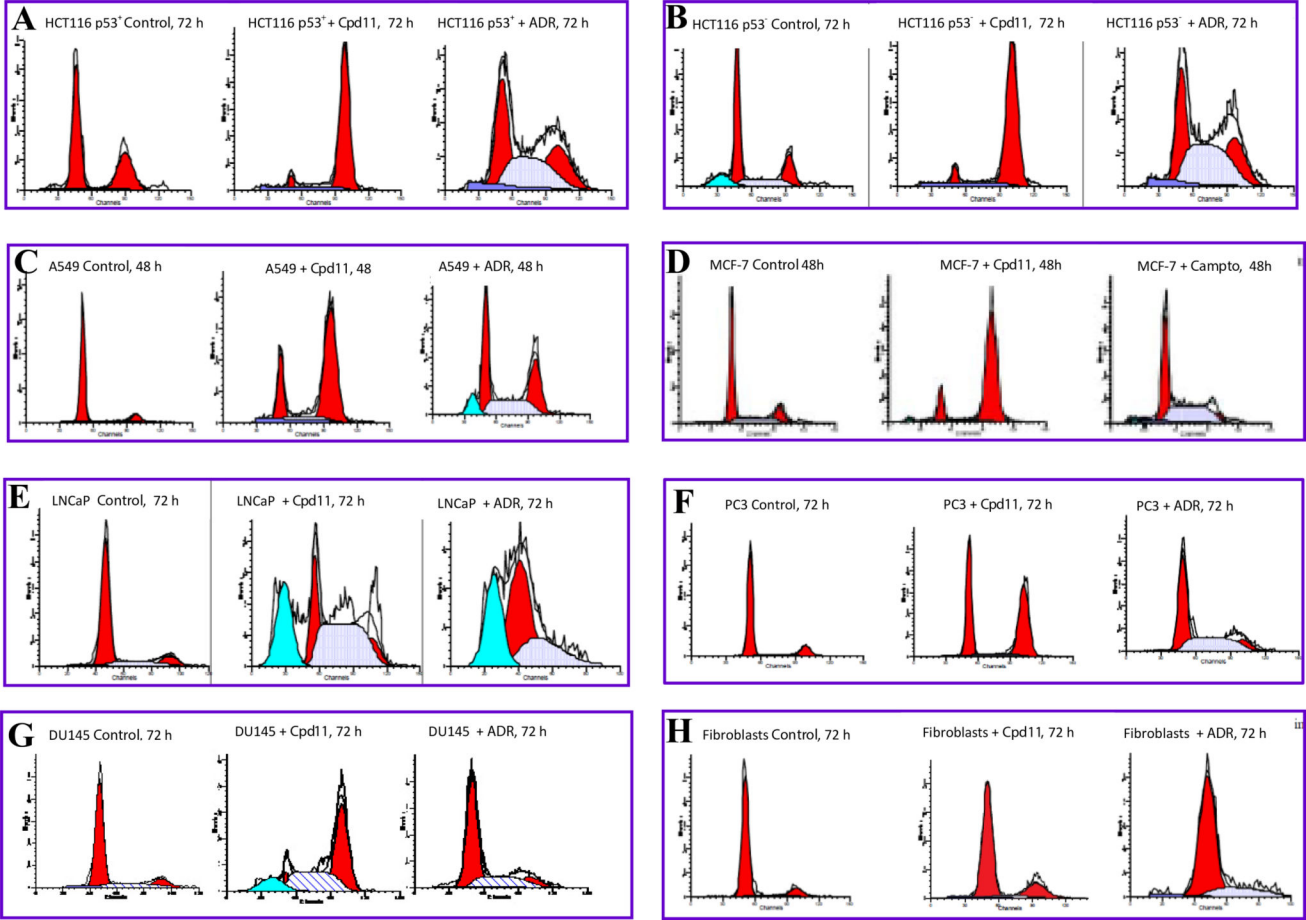
Representative flow cytometric histograms showing effects on various human cancer cell cycles or the cell cycle of normal cells upon treatment with compound **11** (**Cpd11**). The panels are: (A), HCT116 p53 positive (p53^+^) colon cancer cell line; (B), HCT116 p53 negative (p53^−^) colon cancer cell line; (C), A549 lung cancer cell line; (D), MCF-7 breast cancer cell line; (E), LNCaP prostate cancer cells; (F), PC3 prostate cancer cells; (G), DU145 prostate cancer cells; (H), normal human skin fibrobrasts. Cells were exposed to 10 µM of Cpd11 or vehicle (control) or a chemotherapy agent Adriamycin (**ADR**) or Camptothecin (**Campto**) as standard control. The left red peaks represent cells in the G_0_/G_1_-phase of the cell cycle, the right red peaks represent G_2_/M cells, and the area in between them represents cells in the S phase. The cyan peak indicates sub G0 apoptotic peak.

In initial attempts to gain antiproliferative mechanistic insights, we conducted cell cycle perturbation studies with a variety of aggressive human cancer cell lines as follows: colon cancer p53 positive HCT116p^53+/+^ and p53 negative HCT116^p53−/−^ cells, non-small cell lung cancer A549 cells, prostate cancer LNCaP, DU145 and PC3 cells; and to test cancer cell selectivity, we also tested the effects of selected compounds on the cell cycle of normal human fibroblast cells. The results are presented in Figure 5 and supplemental Figure S1. The results show that compound 11, had a very strong G2/M the cell cycle phase arrest in all the cancer cell lines, and in some cancer cells caused in apoptosis. Similar results were also obtained with other potent compounds such as compounds **1** and **2** (data not shown). The difference is quite remarkable when compared with the effects standard anticancer chemotherapy such as Adriamycin (ADR). Similar effects on cell cycle progression were also evident with compound **1** and compound **2** (designated as compounds **110** and **110-5-M2**, respectively, see supplemental data). This is in stark contrast to Adriamycin (a well-known anticancer drug) which induced a very strong G1 cell cycle arrest of the human fibroblasts within the same time period of 72 h. The effect on cell cycle phase distribution by our compounds differentiates them from other β-carbolines such as harmine, which inhibits cell proliferation in a variety of cancer cell lines, but blocks the cell cycle at the G1 phase [43,44]. Moreover, the results also show that this series has a differences in mechanism(s) of action compared to cancer chemotherapeutic agents of the anthracycline (Adriamycin) and the camptothecin classes, which exhibited cell cycle phase arrest in the G1 phase in contrast to the strong G2/M cell cycle arrest that our compounds caused.

Importantly, there was a strong indication of selectivity towards cancer cells relative to normal cells, as these compounds were no significant effects on the cell cycle of normal fibroblast cells. This is unlike the cancer chemotherapeutic agents tested, particularly, Adriamycin which showed strong G1 phase arrest in the cancer cells and the normal cells alike.

## Conclusion

This study has demonstrated the broad and selective anticancer activity of a new series of β-carbolines with impressive broad-based anticancer activity impinging on many aggressive and difficult to treat cancers such as metastatic pancreatic cancer, triple negative breast cancer, metastatic melanoma, as well as lung cancer, colon cancer and metastatic prostate cancer, with unprecedented submicromolar level antiproliferative activity against cancer cell lines, for this chemical class in general. We have acquired a wide-ranging structure activity relationship that gives insights as to the type of chemical modifications round the core template that will maintain high activity and provides a way forward for lead optimization. Major conclusions regarding the influence antiproliferative activity are a hydrophobic 1-napthyl of closely related aromatic substituent at the β-carboline C1 position, and the activity enhancing methoxy or an oxygen bearing electron donating substituent at the C6 position, with the same substituent at the C7 or C8 position of the β-carboline likely going to decrease anticancer activity. Thus, valuable insights have been obtained to guide lead optimization efforts. Compound 11, which emerged as the most potent compound across the board is being pursued in preclinical models [41,42].

## Experimental

### General

All the chemicals and solvents were purchased from Aldrich and used without further purification. All the reactions were performed under nitrogen atmosphere. TLC monitored progress of all the reaction on silica gel plates (Analtech, Inc.). Fisher scientific Da visil grade 1740 (170–400 mesh) was used for flash chromatography to purify the final products. 1H NMR spectra were recorded on Brucker AR, 300 MHz spectrometer: chemical shifts are expressed in δ values (ppm) reference to the TMS and coupling constants (J values) in Hz. Mass spectral data were determined on a Brucker-HP Esquire-LC spectrometer (ESI-MS); and high resolution mass spectrometry (HRMS) was performed at the instrument core of Northwerstern University, Chicago, IL. Where HRMS data are provided, they are the observed exact mass, and the formular for which it was calculated. Unless otherwise noted, the purity of compounds was determined by HPLC analysis using a SUPELCOSIL^®^ 5µm C-18 reverse phase column (250 × 4.6 mm) at ambient temperature on a Waters^®^ 2695 HPLC system equipped with the 996 photodiode array detector. An isocratic method comprising 5% water (solvent A) and 95% methanol (solvent B) was used. 10 min run time was set at a flow-rate of 1.75 mL/min. The % area used to calculate the purity was calculated by peaks detected at 230 nm or 300 nm. The purity of almost all compounds was found to be in the range 90 to 99.9% as determine by HPLC.

### General procedure for the synthesis of tetrahydro-β-carbolines

The tryptamine derivative (0.524 mmol) and aldehyde (0.63 mmol) were dissolved in THF (20 mL). The reaction mixture was cooled to 0°C. CF_3_COOH (0.2 mL) was then added at 0°C and the reaction mixture was stirrred at 0°C for 1 h. The ice bath was then removed and the reaction mixture was stirred for another 1 h. The reaction mixture was quenched with aqueous saturated NaHCO_3_ (5 mL) and the organic phase separated. The mixture was extracted with ethyl acetate (2 × 10 mL) and the combined organic phase was dried over anhydrous Na_2_SO_4_, filtered and the organic solvents evaporated under reduced pressure to give the crude product. The crude tetrahydro-β–carboline product was directly used for second step without further purification.

### General procedure for the synthesis of β-carbolines from the tetrahydro-β-carboline intermediates

To a solution of the crude tetrahydro-β-carboline in xylene (10 mL) was added 10% Pd/C (50–100 mg) and the mixture refluxed overnight. The reaction mixture was then cooled and filtered through celite and washed with MeOH (5–10 mL). Evaporation of the xylene/MeOH filtrate under reduced pressure yielded a crude β-carboline residue. The crude residue was subjected to flash chromatography (30% ethyl acetate in hexanes) to obtain pure β-carbolines in 50–75% overall yields.

### One-step general micro wave experimental procedure for the synthesis of β-carbolines

Tryptamines (0.62 mmol) and aldehydes (0.75 mmol) were dissolved in 4 mL methylene chloride in round bottomed flask. If tryptamines (hydrochloride salts) were insoluble in methylene chloride in those cases few drops of methanol was added to the mixture to dissolve tryptamines completely to make a homogeneous solution mixture. The catalyst was prepared separately by mixing 21 mg of 10% Pd/C and 500 mg of montmorillonite K-10. This catalyst was added to the above reaction mixture and stirred for nearly 5–10 min and solvents were removed under reduced pressure. The dried solid mixture was transferred to 10 mL microwave reaction vial (CEM vials) and irradiated in a CEM Discover Labmate microwave reactor for 60 min at 150°C using pressure and power below 100 psi and 100 W, respectively for all reactions. After the completion of reaction, methylene chloride was added or in some cases (where tryptamine hydrochloride was used) a mixture of methanol and ethyl acetate was added and filtered through celite. The filtrate was concentrated under reduced pressure and the crude residue was subjected to the flash column chromatography using ethyl acetate and hexane solvent mixture (3:7) to obtain β-carbolines in 22–60% yields.

### 7-Methoxy-1-(quinolin-4-yl)-9H-pyrido[3,4-b]indole (1)

Yield 55%; mp: 211–215°C; ^1^H NMR (CDCl_3_): δ 11.45 (bs, 1H, NH), 8.90 (d, J=8.7 Hz, 1H, ArH), 8.71 (s, 1H, ArH), 8.54-8.42 (m, 2H, ArH), 8.22 (d, J=8.7Hz, 1H, ArH), 8.02-7.86 (m,2H, ArH), 7.10 (s, 1H, ArH), 6.84 (dd, J=2.4 and 2.4 Hz, 1H, ArH) and 3.81 (s, 3H, OCH_3_); MS (ESI); *m/z* 325.1 [M−H]^−^.

### 6-Methoxy-1-(quinolin-4-yl)-9H-pyrido[3,4-b]indole (2)

Yield 53%; mp: 228–230°C; ^1^H NMR (CDCl_3_): δ 9.66 (bs, 1H, NH), 8.76 (d, J=4.5 Hz, 1H, ArH), 8.65 (d, J=5.4 Hz, 1H, ArH), 8.10 (d, J=5.4 Hz, 1H, ArH), 8.04 (d, J=8.7 Hz, 1H, ArH), 7.80 (d, J=8.7 Hz, 1H, ArH), 7.70-7.59 (m, 3H, ArH), 7.51-7.44 (m, 3H, ArH) and 4.00 (s, 3H, OCH3); MS (ESI); *m/z* 348.1 [M+Na]^+^.

### 1-(isoquinolin-1-yl)-7-methoxy-9H-pyrido[3,4-b]indole (3)

Yield 55%; 234–235°C; ^1^H NMR (CDCl_3_): δ 11.86 (bs, 1H, NH), 8.58 (d, J=5.5 Hz, 1H, ArH), 8.28 (d, J=4.2Hz, 1H, ArH), 8.14 (d, J=8.7 Hz, 1H, ArH), 8.04 (d, J=5.1 Hz, 1H, ArH), 7.75-7.68 (m, 2H, ArH), 7.44-7.30 (m, 3H, ArH), 7.12 9s, 1H, ArH), 7.01 (d, J=8.7 Hz, 1H, ArH) and 3.90 (s, 3H, OCH_3_); MS (ESI); *m/z* 348.1 [M+Na]^+^.

### 6-Methoxy-1-(quinoxalin-5-yl)-9H-pyrido[3,4-b]indole (4)

Yield 55%; ^1^H NMR (CDCl_3_): δ 8.93 (s, 2H, ArH), 8.73 (d, J=1.8 Hz, 2H, ArH), 8.65 (d, J=5.1 Hz, 1H, ArH), 8.56 (dd, J=1.8 and 1.8 Hz, 1H, ArH), 8.00 (d, J=85.4 Hz, 1H, ArH), 7.64 (d, J=2.4 Hz, 1H, ArH), 7.48 (d, J=9.0 Hz, 1H, ArH), 7.27 (s, 1H, ArH), and 3.98 (s, 3H, OCH_3_); MS (ESI); *m/z* 325.1 [M−H]^−^; HRMS *m/z* 327.1245 [M+H]^+^.

### 7-Methoxy-1-(quinoxalin-5-yl)-9H-pyrido[3,4-b]indole (5)

Yield 55%; mp: 211–215°C; ^1^H NMR (CDCl_3_): δ 11.45 (bs, 1H, NH), 8.90 (d, J=8.7 Hz, 1H, ArH), 8.71 (s, 1H, ArH), 8.54-8.42 (m, 2H, ArH), 8.22 (d, J=8.7Hz, 1H, ArH), 8.02-7.86 (m, 2H, ArH), 7.10 (s, 1H, ArH), 6.84 (dd, J=2.4 and 2.4 Hz, 1H, ArH) and 3.81 (s, 3H, OCH_3_); MS (ESI); *m/z* 325.1 [M−H]^−^.

### 1-(Furan-3-yl)-6-methoxy-9H-pyrido[3,4-b]indole (6)

Yield 60%; mp: 153–155°C; ^1^H NMR (CDCl_3_): δ 8.53 (bs, 1H, NH), 8.48 (d, J=5.1 Hz, 1H, ArH), 8.14 (s, 1H, ArH), 7.85 (d, J=75.4 Hz, 1H, ArH), 7.61 (s, 1H, ArH), 7.57 (s, 1H, ArH), 7.42 (d, J=9.0 Hz, 1H, ArH), 7.21 (dd, J=2.4, 2.4 Hz, 1H, ArH), 7.11 (s, 1H, ArH), 3.95 (s, 3H, OCH_3_), MS (ESI); *m/z* 265.0 [M+H]^+^.

### 1-(Isoquinolin-4-yl)-6-methoxy-9H-pyrido[3,4-b]indole (7)

Yield 47%; mp: 238–240°C; ^1^H NMR (CDCl_3_): δ 10.62(bs, 1H, NH), 8.82 (s, 1H, ArH), 8.76 (s, 1H, ArH), 8.61(d, J=4.5 Hz, 1H, ArH), 8.04 (d, J=5.4 Hz, 1H, ArH), 7.96 (d, J=7.5Hz, 1H, ArH), 7.81-7.60 (m, 4H, ArH), 7.42-7.17 (m, 2H, ArH) and 3.96 (s, 3H, OCH_3_); MS (ESI); *m/z* 324.0 [M−H]^−^.

### 1-(Isoquinolin-4-yl)-7-methoxy-9H-pyrido[3,4-b]indole (8)

Yield 49%; mp: 234–235°C; ^1^H NMR (CDCl_3_): δ 11.67 (bs, 1H, NH), 8.78 (s, 1H, ArH), 8.56 (d, J=5.1 Hz, 1H, ArH), 8.49 (s, 1H, ArH), 8.12-7.92 (m, 2H, ArH), 7.79-7.58 (m, 3H, ArH), 7.87–7.96 (m, 2H, ArH) and 3.81 (s, 3H, OCH_3_); MS (ESI); *m/z* 324.1 [M−H]^−^.

### 6-Methoxy-1-(quinolin-3-yl)-9H-pyrido[3,4-b]indole (9)

Yield 45%; mp: 263–264°C; ^1^H NMR (DMSO-d6): δ 11.67 (bs, 1H, NH), 9.53 (s, 1H, ArH), 8.97 (s, 1H, ArH), 8.52 (d, J=5.1 Hz, 1H, ArH), 8.27-8.10 (m, 3H, ArH), 7.92-7.80 (m, 2H, ArH), 7.29 (t, J=6.9 Hz, 1H, ArH), 7.58 (d, J=9.0 Hz, 1H, ArH), 7.24 (d, J=9.0 Hz, 1H, ArH) and 3.89 (s, 3H, OCH_3_); MS (ESI); *m/z* 324.0 [M−H]^−^.

### 7-Methoxy-1-(quinolin-3-yl)-9H-pyrido[3,4-b]indole (10)

Yield 52%; mp: 148–151°C; ^1^H NMR (DMSO-d6): δ 9.58 (s, 1H, ArH), 8.82 (s, 1H, ArH), 8.52 (d, J=5.1 Hz, 1H, ArH), 8.16 (d, J=8.4 Hz, 1H, ArH), 8.00 (d, J=8.7 Hz, 1H, ArH), 7.90 (d, J=5.1 Hz, 1H, ArH), 7.29 (t, J=7.2 Hz, 1H, ArH), 7.63 (t, J=7.2 Hz, 1H, ArH), 7.55 (s, 1H, ArH), 7.07 (s, 1H, ArH), 6.90 (d, J=8.4 Hz, 1H, ArH) and 3.91 (s, 3H, OCH_3_); MS (ESI); *m/z* 324.0 [M−H]^−^.

### 6-Methoxy-1-(naphthalen-1-yl)-9H-pyrido[3,4-b]indole (11)

Yield 54%; mp: 115–117°C; ^1^H NMR (CDCl3): δ 8.56 (d, J=11.4 Hz, 1H, ArH), 8.21 (bs, 1H, NH), 8.04-7.87(m, 3H, ArH), 7.81-7.69 (m, 2H, ArH), 7.65-7.37 (m, 4H, ArH), 7.23-7.10 (m, 2H, ArH) and 3.95 (s, 3H, OCH_3_); MS (ESI); *m/z* 323.0 [M−H]^−^.

### 7-Methoxy-1-(naphthalen-1-yl)-9H-pyrido[3,4-b]indole (12)

Yield 59%; mp: 222–225°C; ^1^H NMR (CDCl_3_): δ 8.58 (d, J=5.4 Hz, 1H, ArH), 8.09 (bs, 1H, NH), 8.07-7.90 (m, 4H, ArH), 7.76 (t, J=8.4 Hz, 2H, ArH), 7.65-7.40 (m, 3H, ArH), 6.92 (d, J=8.7 Hz, 1H, ArH), 6.76 (s, 1H, ArH) and 3.83 (s, 3H, OCH_3_); MS (ESI); *m/z* 323.0 [M−H]^−^.

### 6-Methoxy-1-(quinolin-5-yl)-9H-pyrido[3,4-b]indole (13)

Yield 59%; mp: 252–255°C; ^1^H NMR (DMSO-d6): δ 11.00 (bs, 1H, NH), 8.94 (s, 1H, ArH), 8.48 (d, J=5.1 Hz, 1H, ArH), 8.19 (d, J=8.7 Hz, 2H, ArH), 8.05-7.85 (m, 3H, ArH), 7.53-7.38 (m, 2H, ArH), 7.16 (d, J=8.7 Hz, 1H, ArH), and 3.88 (s, 3H, OCH_3_); MS (ESI); *m/z* 324.0 [M− H]^−^.

### 7-Methoxy-1-(quinolin-5-yl)-9H-pyrido[3,4-b]indole (14)

Yield 56%; mp: 232–236°C; ^1^H NMR (DMSO-d6): δ 8.97 (s, 1H, ArH), 8.60 (d, J=5.4 Hz, 1H, ArH), 8.30-8.20 (m, 2H, ArH), 8.06 (d, J=8.7 Hz, 2H, ArH), 8.00-7.90 (m, 2H, ArH), 7.32 (dd, J=4.2 and 4.2 Hz, 1H, ArH), 6.96 (s, 1H, ArH), 6.89 (dd, J=2.1 and 2.1 Hz, 1H, ArH) and 3.90 (s, 3H, OCH_3_); MS (ESI); *m/z* 324.0 [M−H]^−^.

### 1-(Isoquinolin-5-yl)-6-methoxy-9H-pyrido[3,4-b]indole (15)

Yield 59%; mp: 148–150°C; ^1^H NMR (CDCl_3_): δ 9.65 (bs, 1H, NH), 8.85 (s, 1H, ArH), 8.56 (d, J=5.4 Hz, 1H, ArH), 8.24 (d, J=6.0 Hz, 1H, ArH), 8.05-7.85 (m, 3H, ArH), 7.75-7.63 (m, 3H, ArH), 7.49 (d, J=6.0 Hz, 1H, ArH), 7.35 (d, J=9.0 Hz, 1H, ArH), 7.18 (dd, J=2.4 and 2.4 Hz, 1H, ArH) and 3.96 (s, 3H, OCH_3_); MS (ESI); *m/z* 323.9 [M−H]^−^.

### 1-(Isoquinolin-5-yl)-7-methoxy-9H-pyrido[3,4-b]indole (16)

Yield 70%; mp: 140–142°C; ^1^H NMR (CDCl_3_): δ 9.20 (s, 1H, ArH), 8.62 (d, J=5.4 Hz, 1H, ArH), 8.53 (bs, 1H, NH), 8.42 (d, J=6.0 Hz, 1H, ArH), 8.20-8.03 (m, 3H, ArH), 7.80 (t, J=8.1Hz, 1H, ArH), 7.62 (d, J=6.0 Hz, 1H, ArH), 6.96 (dd, J=2.4 and 2.4 Hz, 1H, ArH), 6.89 (s, 1H, ArH) and 3.88 (s, 3H, OCH_3_); MS (ESI); *m/z* 323.9 [M−H]^−^.

### 6-Methoxy-1-(naphthalen-2-yl)-9H-pyrido[3,4-b]indole (17)

Yield 67%; ^1^H NMR (CDCl_3_ and DMSO-d6): δ 8.58-8.43 (m, 2H, ArH), 8.14 (d, J=8.4 Hz, 1H, ArH), 8.06-7.885 (m, 4H, ArH), 7.64-7.46 (m, 4H, ArH), 8.16 (d, J=8.1 Hz, 1H, ArH), and 3.92 (s, 3H, OCH_3_); MS (ESI); *m/z* 323.1 [M−H]^−^.

### 7-Methoxy-1-(naphthalen-2-yl)-9H-pyrido[3,4-b]indole (18)

Yield 60%; mp: 195–196°C; ^1^H NMR (CDCl_3_): δ 8.78 (bs, 1H, NH), 8.56 (d, J=5.4 Hz, 1H, ArH), 8.35 (s, 1H, ArH), 8.12-7.97 (m, 3H, ArH), 7.96-7.83 (m, 3H, ArH), 7.60-7.48 (m, 2H,ArH), 6.96 (s, 1H, ArH) and 3.90 (s, 3H, OCH_3_); MS (ESI); *m/z* 323.2 [M−H]^−^.

### 1-(Anthracen-9-yl)-6-methoxy-9H-pyrido[3,4-b]indole (19)

Yield 59%; mp: 215–219°C; ^1^H NMR (CDCl_3_): δ 8.72 (d, J=5.1 Hz, 1H, ArH), 8.64 (s, 1H, NH), 8.20-8.08 (m, 3H, ArH), 7.68 (s, 1H, ArH), 7.62 (s, 1H, ArH), 7.53-7.45 (m, 3H, ArH), 7.39-7.27 (m, 2H, ArH), 7.13 (s, 2H, ArH) and 3.97 (s, 3H, OCH_3_); MS (ESI); *m/z* 373.0 [M−H]^−^.

### 1-(Anthracen-9-yl)-7-methoxy-9H-pyrido[3,4-b]indole (20)

Yield 63%; mp: 270–271°C; ^1^H NMR (CDCl_3_): δ 9.49 (s, 1H, NH), 8.70-8.59 (m, 2H, ArH), 8.15-8.00 (m, 4H, ArH), 7.55-7.40 (m, 4H, ArH), 7.30 (d, J=7.8 Hz, 2H, ArH), 6.77 (dd, J=2.1 and 2.1 Hz, 1H, ArH), 6.78 (s, 3H, ArH) and 3.79 (s, 3H, OCH_3_); MS (ESI); *m/z* 373.1 [M−H]^−^.

### 6-Methoxy-1-(phenanthren-9-yl)-9H-pyrido[3,4-b]indole (21)

Yield 55%; mp: 240–242°C; ^1^H NMR (CDCl_3_): δ 8.79 (t, J=8.7 Hz, 2H, ArH), 8.55 (d, J=5.1 Hz, 1H, ArH), 8.05-7.94 (m, 3H, ArH), 7.81 (d, J=8.1Hz, 1H, ArH), 7.76-7.61 (m, 4H, ArH), 7.49 (t, J=7.8 Hz, 2H, ArH), 7.36 (d, J=8.7 Hz, 1H, ArH), 7.13 (dd, J=2.4 and 2.4 Hz, 1H, ArH) and 3.93 (s, 3H, OCH3); MS (ESI); *m/z* 373.0 [M−H]^−^.

### 7-Methoxy-1-(phenanthren-9-yl)-9H-pyrido[3,4-b]indole (22)

Yield 61%; mp: 155–156°C; ^1^H NMR (CDCl_3_): δ 8.80 (d, J=8.1 Hz, 1H, ArH), 8.75 (d, J=8.1 Hz, H, ArH), 8.58 (d, J=5.4 Hz, 1H, ArH), 8.17 (s, 1H, NH), 8.08-7.88 (m, 4H, ArH), 7.82-7.60 (m, 4H, ArH), 7.50 (t, J=7.8 Hz, 2H, ArH), 6.92 (dd, J=2.1 and 2.1 Hz, 1H, ArH), 6.73 (s, 1H, ArH) and 3.80 (s, 3H, OCH_3_); MS (ESI); *m/z* 373.0 [M−H]^−^.

### 6-Methoxy-1-phenyl-9H-pyrido[3,4-b]indole (23)

Yield 51%; ^1^H NMR (CDCl_3_): δ 8.81 (bs, 1H, NH), 8.52 (d, J=5.4 Hz, 1H, ArH), 7.97-7.86 (m, 3H, ArH), 7.58 (s, 1H, ArH), 7.55-7.32 (m, 4H, ArH), 7.19 (d, J=9.0 Hz, 1H, ArH) and 3.94 (s, 3H, OCH_3_); MS (ESI); *m/z* 273.0 [M−H]^−^.

### 7-Methoxy-1-phenyl-9H-pyrido[3,4-b]indole (24)

Yield 55%; mp: 225–227°C; ^1^H NMR (CDCl3): δ 8.54 (d, J=5.4 Hz, 2H, ArH), 8.06-7.93 (m, 3H, ArH), 7.84 (d, J=5.1 Hz, 1H, ArH), 7.58 (t, J=7.8 Hz, 2H, ArH), 7.48 (t, J=7.6 Hz, 1H, ArH), 6.97-6.92 (m, 2H, ArH) and 3.91 (s, 3H, OCH3); MS (ESI); m/z 273.0 [M−H]^−^.

### 6-Methoxy-1-(2-methylnaphthalen-1-yl)-9H-pyrido[3,4-b]indole (25)

Yield 50%; mp: 212–214°C; ^1^H NMR (CDCl_3_): δ 8.59 (d, J=5.4 Hz, 1H, ArH), 7.80 (d, J=5.4 Hz, 1H, ArH), 8.91 (d, J=8.4 Hz, 2H, ArH), 7.82 (bs, 1H, NH), 7.65 (s, 1H, ArH), 7.54-7. 40 (m, 2H, ArH), 7.34-7.12 (m, 4H, ArH) and 3.96 (s, 3H, OCH_3_); MS (ESI); *m/z* 339.2 [M+H]^+^.

### 7-Methoxy-1-(2-methylnaphthalen-1-yl)-9H-pyrido[3,4-b]indole (26)

Yield 55%; mp: 213–214°C; ^1^H NMR (CDCl_3_): δ 8.53 (d, J=5.4 Hz, 1H, ArH), 7.05 (d, J=5.4 Hz, 1H, ArH), 8.01 (bs, 1H, NH), 7.94-7. 85 (m, 3H, ArH), 7.51-7.37 (m, 2H, ArH), 7.31-7.21 (m, 2H, ArH), 6.90 (dd, J=2.4 and 2.4 Hz, 1H, ArH), 6.65 (s, 1H, ArH) and 3.75 (s, 3H, OCH_3_); MS (ESI); *m/z* 339.2 [M+H]^+^.

### 6-Methoxy-1-(6-methoxynaphthalen-1-yl)-9H-pyrido[3,4-b]indole (27)

Yield 50%; mp: 213–217°C; ^1^H NMR (CDCl_3_): δ 8.69 (bs, 1H, NH), 8.57 (d, J=5.4 Hz, 1H, ArH), 8.31 (s, 1H, ArH), 8.05 (dd, J=1.5 and 1.5Hz, 1H, ArH), 7.92-7.79 (m, 3H, ArH), 7.61(s, 1H, ArH), 7.43 (d, J=9.0 Hz, 1H, ArH), 7.30-7.15 (m, 3H, ArH), 3.97 (s, 3H, OCH_3_) and 3.96 (s, 3H, OCH3); MS (ESI); *m/z* 353.0 [M−H]^−^.

### 6-(Benzyloxy)-1-(naphthalen-1-yl)-9H-pyrido[3,4-b]indole (28)

Yield 72%; ^1^H NMR (CDCl_3_): δ 8.59 (d, J=5.4 Hz, 1H, ArH), 8.10-7.96 (m, 4H, ArH), 7.81-7.71 (m, 3H, ArH), 7.65-7.54 (m, 4H, ArH), 7.48-7.35 (m, 4H, ArH), 7.23 (s, 1H, ArH) and 5.22 (s, 2H, OCH2); MS (ESI); *m/z* 401.0 [M+H]^+^.

### 8-Methyl-1-(naphthalen-1-yl)-9H-pyrido[3,4-b]indole (29)

Yield 72%; ^1^H NMR (CDCl_3_): δ 8.67(d, J=5.1 Hz, 1H, ArH), 8.12-7.98 (m, 3H, ArH), 7.86-7.67 (m, 3H, ArH), 7.57 (t, J=6.9 Hz, 1H, ArH), 7.45 (t, J=6.9 Hz, 1H, ArH), 7.36 (d, J=7.2 Hz,1H, ArH), 7.30-7.25 (m, 2H, ArH) and 2.43(s, 3H, CH_3_); MS (ESI); *m/z* 307.1 [M−H]^−^.

### 6-Methoxy-1-(4-(pyridin-2-yl)phenyl)-9H-pyrido[3,4-b]indole (30)

Yield 42%; mp: 189–190°C; ^1^H NMR (CDCl_3_): δ 9.21 (bs, 1H, NH), 8.70 (s, 1H, ArH), 8.52 (d, J=5.1 Hz, 1H, ArH), 8.10-7.86 (m, 5H, ArH), 7.78-7.54 (m, 3H, ArH), 7.40 (d, J=8.7 Hz, 1H, ArH), 7.30-7.15 (m, 2H, ArH) and 3.94 (s, 3H, OCH_3_); MS (ESI); *m/z* 352.3[M+H]^+^.

### 8-methoxy-1-(naphthalen-1-yl)-9H-pyrido[3,4-b]indole (31)

Yield 28%; ^1^H NMR (CDCl_3_): δ 8.63 (d, J=5.0 Hz, 1H, ArH), 8.13 (s, 1H, ArH), 8.08-7.97 (m, 2H, ArH), 7.83-7.64 (m, 3H, ArH), 7.59-7.51 (m, 2H, ArH), 7.48-7.40 (m, 1H, ArH), 7.27-7.21(m, 1H, ArH), 6.98 (d, J=6.0 Hz, 1H, ArH), and 3.90 (s, 3H, CH_3_); MS (ESI); m/z 325.1 [M+H]^+^; HRMS; *m/z* 325.1342 [M+H]^+^; calculated for C_22_H_16_N_2O_.

### 1-(naphthalen-1-yl)-9H-pyrido[3,4-b]indol-6-ol (32)

Yield 26%; ^1^H NMR (CDCl_3_): δ 8.64 (d, J=6.0 Hz, 1H, ArH), 8.08-7.94 (m, 2H, ArH), 7.86-7.75 (m, 2H, ArH), 7.70-7.42 (m, 5H, ArH), 7.25 (d, J=9.0 Hz, 1H, ArH) and 7.10 (d, J=9.0 Hz, 1H, ArH); MS (ESI); *m/z* 311.1 [M+H]^+^.

### 6-methoxy-1-(naphthalen-1-yl)-4-phenyl-9H-pyrido[3,4-b]indole (33)

Yield 35%; ^1^H NMR (CDCl_3_): δ 8.52 (s, 1H, ArH), 8.08-7.78 (m, 5H, ArH), 7.74-7.45 (m, 6H, ArH), 7.38-7.33 (m, 2H, ArH), 7.08-7.22 (m, 2H, ArH) and 3.7 (s, 3H, CH_3_); MS (ESI); *m/z* 401.1 [M+H]^+^; HRMS *m/z* 401.1657 [M+H]^+^.

### N-methyl-1-(1-(naphthalen-1-yl)-9H-pyrido[3,4-b]indol-6-yl)methanesulfonamide (34)

Yield 24%; ^1^H NMR (CDCl_3_): δ 8.70 (d, J=5.5 Hz, 1H, ArH), 8.23 (s, 1H, ArH), 8.05-7.98 (m,3H, ArH), 7.79 (d, J=5.5 Hz, 1H, ArH), 7.74 (d, J=8.5 Hz, 1H, ArH), 7.68-7.65 (m, 1H, ArH), 7.56-7.53 (m, 2H, ArH), 7.46-7.38 (m, 2H, ArH), 4.45 (s, 2H, CH_2_) and 2.76 (d, J=5.5 Hz, 3H, CH_3_); MS (ESI); *m/z* 402.1 [M+H]^+^.

### 5-Methyl-1-(naphthalen-1-yl)-9H-pyrido[3,4-b]indole (35)

Yield 26%; ^1^H NMR (CDCl_3_): δ 8.67 (d, J=5.6 Hz, 1H, ArH), 8.12 (d, J=5.6 Hz, 1H, ArH), 8.03-7.98 (m, 2H, ArH), 7.79 (d, J=7.2 Hz, 1H, ArH), 7.74 (d, J=8.4 Hz, 1H, ArH), 7.68-7.64 (m, 1H, ArH), 7.56-7.52 (m, 2H, ArH), 7.43-7.39 (m, 2H, ArH), 7.22 (d, J=8.0 Hz, 1H, ArH), 7.09 (d, J=7.6 Hz, 1H, ArH) and 2.95 (s, 3H, CH_3_); MS (ESI); *m/z* 309.1 [M+H]^+^.

### 1-(5-fluoronaphthalen-1-yl)-6-methoxy-9H-pyrido[3,4-b]indole (36)

Yield 28%; ^1^H NMR (CDCl_3_): δ 8.64 (d, J=5.4 Hz, 1H, ArH), 8.32 (d, J=8.7 Hz, 1H, ArH), 8.02 (d, J=5.4 Hz, 1H, ArH), 7.90-7.82 (m, 2H, ArH), 7.76-7.55 (m, 3H, ArH), 7.43-7.18 (m, 3H, ArH) and 3.98 (s, 3H, OCH_3_); MS (ESI); *m/z* 340.9 [M−H]^−^; HRMS; *m/z* 343.1249; calculated for C_22_H_15_FN_2O_.

### 1-(5-bromonaphthalen-1-yl)-6-methoxy-9H-pyrido[3,4-b]indole (37)

Yield 30%; ^1^H NMR (CDCl_3_): δ 8.60 (d, J=5.6 Hz, 1H, ArH), 8.44 (d, J=5.0 Hz, 1H, ArH), 7.01 (d, J=5.0 Hz, 1H, ArH), 7.87-7.71 (m, 3H, ArH), 7.63 (s, 1H, ArH), 7.28-7.16 (m, 3H, ArH) and 3.98 (s, 3H, CH_3_); MS (ESI); *m/z* 403.1 and 404.1 (bromo pattern) [M+H]^+^; HRMS; *m/z* 404.0484 [M+H]^+^; calculated for C_22_H_15_BrN_2O_.

### 1-(5-bromonaphthalen-1-yl)-6-methyl-9H-pyrido[3,4-b]indole (38)

Yield 24%; ^1^H NMR (CDCl_3_): δ 8.64 (d, J=5.0 Hz, 1H, ArH), 8.45 (d, J=8.5 Hz, 1H, ArH), 8.02-7.98 (m, 2H, ArH), 7.85-7.72 (m, 3H, ArH), 7.57 (t, J=6.9 Hz, 1H, ArH), 7.56-7.53 (m, 1H, ArH), 7.36 (d, J=8.0 Hz, 1H, ArH), 7.28-7.24 (m, 2H, ArH) and 2.58 (s, 3H, CH3); MS (ESI); *m/z* 384.6 and 385.6 (bromo pattern) [M−H]^−^; HRMS *m/z* 387.0493 [M+H]^+^; calculated for C_22_H_15_BrN_2_.

### 1-(1,2-dihydroacenaphthylen-5-yl)-6-methoxy-9H-pyrido[3,4-b]indole (39)

Yield 23%; ^1^H NMR (CDCl_3_): δ 8.60 (d, J=5.0 Hz, 1H, ArH), 8.01 (bs, 1H, NH), 7.95 (d, J=5.5 Hz, 1H, ArH), 7.79 (d, J=7.5 Hz, 1H, ArH), 7.61 (s, 1H, ArH), 7.56 (d, J=8.5 Hz, 1H, ArH), 7.45-7.39 (m, 2H, ArH), 7.34 (d, J=6.5 Hz, 1H, ArH), 7.25 (s, 1H, ArH), 7.15 (d, J=9.0 Hz, 1H, ArH), 3.94 (s, 3H, OCH_3_) and 3.48 (s, 4H, CH_2_); MS (ESI); *m/z* 350.41 [M+H]^+^; HRMS *m/z* 351.1497 [M+H]^+^; calculated for C_24_H_18_N_2O_.

### 6-Chloro-1-(naphthalen-1-yl)benzo[4,5]thieno[2,3-c] pyridine (40)

Yield 32%; ^1^H NMR (CDCl_3_): δ 8.87 (d, J=5.5 Hz, 1H, ArH), 8.26 (s, 1H, ArH), 8.04-7.99 (m, 2H, ArH), 7.95 (d, J=8.5 Hz, 1H, ArH), 7.81-7.71 (m, 3H, ArH), 7.61 (t, J=8.0 Hz, 1H, ArH), 7.52 (t, J=7.0 Hz, 2H, ArH) and 7.42 (t, J=8.0 Hz, 1H, ArH); MS (ESI); *m/z* 346.1 [M+H]^+^; HRMS *m/z* 346.0456 [M+H]^+^, Calculated for C_21_H_12_ClNS.

### 1-(Naphthalen-1-yl)benzofuro[2,3-c]pyridine (41)

Yield 22%; ^1^H NMR (CDCl_3_): δ 8.77 (d, J=5.0 Hz, 1H, ArH), 8.10 (d, J=7.5 Hz, 1H, ArH), 8.02 (d, J=8.5 Hz, 1H, ArH), 7.96 (d, J=5.0 Hz, 2H, ArH), 7.91 (d, J=8.5 Hz, 1H, ArH), 7.86 (d, J=6.5 Hz, 1H, ArH), 7.65 (t, J=8.0 Hz, 1H, ArH), 7.62-7.50 (m, 3H, ArH) and 7.44 (t, J=8.0 Hz, 2H, ArH); MS (ESI); *m/z* 296.7 [M+H]^+^; HRMS *m/z* 296.1075 [M+H]^+^; calculated for C_21_H_13_NO.

### 1-(Naphthalen-1-yl)benzo[4,5]thieno[2,3-c]pyridine (42)

Yield 31%; ^1^H NMR (CDCl_3_): δ 8.83 (d, J=5.5 Hz, 1H, ArH), 8.27 (d, J=7.0 Hz, 1H, ArH), 8.04 (d, J=5.5 Hz, 1H, ArH), 7.99 (d, J=8.5 Hz, 1H, ArH), 7.94 (d, J=8.0 Hz, 1H, ArH), 7.79 (t, J=8.0 Hz, 3H, ArH), 7.60 (t, J=7.5 Hz, 1H, ArH), 7.58-7.48 (m, 3H, ArH) and 7.40 (t, J=7.5 Hz, 1H, ArH); MS (ESI); *m/z* 312.4 [M+H]^+^; HRMS *m/z* 312.0849 [M+H]^+^; calculated for C_21_H_13_NS.

### 1-(Naphthalen-1-yl)-9H-pyrido[3,4-b]indole (43)

Yield 28%; ^1^H NMR (CDCl_3_): δ 8.66 (d, J=5.4 Hz, 1H, ArH), 8.28-7.95 (m, 4H, ArH), 7.78 (t, J=6.9 Hz, 2H, ArH), and 7.68-7.26 (m, 6H, ArH); MS (ESI); *m/z* 293.0 [M−H]^−^.

### 6-Methyl-1-(naphthalen-1-yl)-9H-pyrido[3,4-b]indole (44)

Yield 34%; ^1^H NMR (CDCl_3_): δ 8.61 (d, J=5.0 Hz, 1H, ArH), 8.05-7.54 (m, 2H, ArH), 7.90 (s, 1H, ArH), 7.76 (t, J=9.0 Hz, 2H, ArH), 7.63 (t, J=7.5 Hz, 1H, ArH), 7.52 (t, J=7.5 Hz, 1H, ArH), 7.40 (t, J=7.5 Hz, 1H, ArH), 7.32 (d, J=8.0 Hz, 1H, ArH), 7.60-7.15 (m, 2H, ArH) and 2.54 (s, 3H, CH3); MS (ESI); m/z 309.9 [M+H]^+^; HRMS *m/z* 309.1393 [M+H]^+^; calculated for C_22_H_16_N_2_.

### 1-(Naphthalen-1-yl)-6-(trifluoromethoxy)-9H-pyrido[3,4-b]indole (45)

Yield 34%; ^1^H NMR (CDCl_3_): δ 8.62 (d, J=5.2 Hz, 1H, ArH), 8.34 (s, 1H, ArH), 8.10-7.92 (m, 3H, ArH), 7.73 (d, J=7.4 Hz, 2H, ArH) and 7.65-7.25 (m, 5H, ArH); MS (ESI); *m/z* 379.9 [M+H]^+^.

### 1-(naphthalen-1-yl)-9H-pyrido[3,4-b]indole-6-carbonitrile (46)

Yield 32%; ^1^H NMR (CDCl_3_): δ 8.77 (d, J=6.0 Hz, 1H, ArH), 8.56 (s, 1H, ArH), 7.90-7.82 (m, 2H, ArH), 8.09-8.00 (m, 3H, ArH), 7.80-7.73 (m, 3H, ArH), 7.57-7.53 (m, 2H, ArH) and 7.48-7.44 (m, 2H, ArH); MS (ESI); *m/z* 321.1 [M+H]^+^; HRMS *m/z* 320.1184 [M+H]^+^; calculated for C_22_H_13_N_3_.

### Ethyl 6-methoxy-1-(naphthalen-1-yl)-9H-pyrido[3,4-b]indole-3-carboxylate (47)

Yield 25%; ^1^H NMR (CDCl_3_): δ 8.92 (s, IH, NH), 8.14 (s, 1H, ArH), 8.10-7.93 (m, 2H, ArH), 7.78 -7.58 (m, 3H, ArH), 7.54-7.36 (m, 3H, ArH), 7.30-7.24 (m, 2H, ArH), 7.18 (d, J=9.0 Hz, 1H, ArH), 4.50 (q, J=7.0 Hz, 2H, OCH_2_), 3.96 (s, 3H, OCH_3_) and 1.46 (t, J=7.0 Hz, 3H, CH_3_); MS (ESI); m/z 397.3 [M+H]^+^; HRMS *m/z* 397.1552 [M+H]^+^, Calculated for C_25_H_20_N_2_O_3_

### 9-Methyl-1-(naphthalen-1-yl)-9H-pyrido[3,4-b]indole (48)

Yield 21%; ^1^H NMR (CDCl_3_): δ 8.60 (d, J=4.8 Hz, 1H, ArH), 8.23 (d, J=7.6 Hz, 1H, ArH), 8.10-7.91 (m, 3H, ArH), 7.70-7.54 (m, 3H, ArH), 7.52-7.27 (m, 5H, ArH) and 3.10 (s, 3H, NCH_3_); MS (ESI); *m/z* 309.1 [M+H]^+^.

### 6-Methoxy-9-methyl-1-(naphthalen-1-yl)-9H-pyrido[3,4-b]indole (49)

Yield 29%; ^1^H NMR (CDCl_3_): δ 8.56 (d, J=5.2 Hz, 1H, ArH), 8.05-7.30 (m, 3H, ArH), 7.69-7.59 (m, 3H, ArH), 7.48 (t, J=7.2 Hz, 1H, ArH), 7.40-7.31 (m, 2H, ArH), 7.27-7.20 (m, 2H, ArH), 3.97 (s, 3H, OCH_3_) and 3.07 (s, 3H, NCH_3_); MS (ESI); m/z 339.3 [M+H]^+^; HRMS *m/z* 339.1501 [M+H]^+^; Calculated for C_23_H_18_N_2_O.

## Molecular Docking Simulations

Docking simulations were carried out with the Schrodinger Inc. modeling suite’s Glide module using crystal structure of the MDM2 protein complex (PDB code: 4ERE) with active inhibitor and using Glide XP docking protocol implemented in the Schrodinger software suite (Schrodinger molecular modeling suite, 2016, Schrodinger Inc., Sand Diego, CA). In the first step protein-ligand complex was prepared by protein preparation wizard. All water compounds were removed from pdb, then hydrogen’s and OPLS_2005 charges [45] were added, structural errors such as missing atoms in side chains (Gln 44) was corrected. Hydrogen bond network optimized and protonation states of amino acid was calculated at pH 7 and later whole protein was restrained minimized to RMSD 0.3 Å for heavy atoms. The receptor grid file was generated using crystal bound ligand OR2 and selecting amino acids within 14Å box. The input ligand structures were drawn using 2D sketcher and converted into 3D using ligprep. In order find out possible role of substituents and structure activity relationship (SAR) using protein-ligand interaction.

## Antiproliferative Assays

The human colon cancer cell line HCT116 and its p53-deficient (HCT116-p53−/−), were kindly provided by Dr. Vogelstein (The Sidney Kimmel Comprehensive Cancer Center, The Johns Hopkins University Medical Institutions, Baltimore) to Dr. Parker Suttle (UTHSC). HCT116 wild-type and p53 mutant lines were maintained at 37°C in a 5% CO2 incubator in McCoy's 5A medium supplemented with 10% fetal bovine serum (FBS), 2 mML-glutamine, and 25 µg/ml gentamicin. Most other human cancer cell lines were obtained from American Type Culture Collection (Rockville, MD, USA) and grown in cell culture media supplemented with 10% fetal bovine serum (FBS) and 1% penicillin/streptomycin (Atlanta Biologicals, Lawrenceville, GA, USA), except for those indicated otherwise. MCF-7 human breast cancer cells were grown in DMEM media, and MDA-MB-468 cells were grown in DMEM/F-12 Ham's media (DMEM/F-12 1:1 mixture). HPAC human pancreatic cells were grown in DMEM/Ham’s F12 media containing 1.2 g/L sodium bicarbonate, 2.5 mM L-glutamine, 15 mM HEPES and 0.5 mM sodium pyruvate supplemented with 2 µg/mL insulin, 5 µg/mL transferrin, 40 ng/mL hydrocortisone, 10 ng/mL epidermal growth factor and 5% FBS; Human Panc-1 cells were cultured in RPMI 1640 containing 1 mM HEPES buffer, 25 µg/mL gentamicin, 1.5 g/L sodium bicarbonate and 0.25 µg/mL amphotericin B; and MIA PaCa-2 pancreatic cancer cells were grown in DMEM containing 2.5% horse serum. Human prostate cancer PC3 cells were maintained in Ham’s F12 medium containing 2 mM L-glutamine; LNCaP human cancer cells were cultured in RPMI 1640 media containing 2 mM L-glutamine, 10 mM HEPES, 1 mM sodium pyruvate, glucose (4.5 mg/mL), and sodium bicarbonate (1.5 mg/mL); and DU145 human prostate cancer cells were cultured in RPMI 1640 medium. Human lung cancer A549 cells were grown in Ham’s F12 medium.

The CellTiter 96^®^ AQueous non-radioactive cell proliferation assay (Promega) was used to detect cell viability. The kit is composed of solutions of a novel tetrazolium compound (3-(4,5-dimethylthiazol-2-yl)-5-(3-carboxymethoxyphenyl)-2-(4-sulfophenyl)-2H-tetrazolium, inner salt (MTS)) and an electron coupling reagent (phenazine methosulfate (MS)). MTS is bioreduced by cells into a formazan product that is soluble in a tissue culture medium. The conversion of MTS into the aqueous soluble formazan product is accomplished by the dehydrogenase enzymes found in metabolically active cells. The quantity of formazan product, as measured by the amount of absorbance at 490 nm is directly proportional to the number of living cells. HCT116 and its p53 deficient cells (1 × 10^4^) were seeded into each well of 96-well plates. The cells were cultured in a CO_2_ incubator until 80% confluence. The cells were then treated with either different doses of compound or DMSO control (0.1% v/v) for 24 h. The medium was then changed and replaced with 100 µl of fresh 10% FBS/ McCoy's 5A medium and 20 µl of MTS solution (Promega). Cells were incubated for another 2 h and 25 µl of 10% SDS was then added to stop the reaction. The optical absorbance was determined using a micro-plate reader (Quant™ BioTek Instruments) at a 490 nm wavelength. The results were presented as percentages of growth inhibition.

The human melanoma A375 cell line, acquired from ATCC, and the human melanoma WM164 cell line, a kind gift from Dr. Meenhard Herlyn (Wistar Institute, Philadelphia, PA), were both cultured in DMEM medium (Mediatech, Inc., VA), supplemented with 10% fetal bovine serum (FBS, Atlanta Biologicals, GA), 1% antibiotic/antimycotic mixture and 5 µg/mL bovine insulin (Sigma-Aldrich, St. Louis, MO). They were then used in the MTS assay as described below to assess the effects of compounds on their viability. In brief, 5000 cells in 100 µL were plated into each well of clear-bottom 96-well cell culture plate overnight. Then the supernatant cell culture medium was carefully aspirated and replaced with 100 µL fresh cell culture medium containing test compounds at serial diluted concentrations. After 48 h incubation, 20 µL 6 × MTS solution prepared per manufacture’s instruction (CellTiter 96R AQueous, Promega Corporation, WI) was added into each well and incubated in dark at 37°C for 1 h. The colorimetric absorbance at 490 nm was determined using a Synergy 2 micro-plate reader (BioTek Instruments, VT). IC_50_ values were calculated from dose-response experiments.

## Cell Cycle Analysis

Analysis of the cell cycle was carried out by flow cytometry. The cells were plated in 6-well plates at 10^6^ cells were harvested, and incubated with 10 µM test compound or DMSO vehicle for control cells. After exposure to the compound for the indicated length of time, 24, 48 or 72 h, the cells were harvested, pelleted and washed with culture medium without serum. Cells were fixed in 1 ml of ice-cold 70% ethanol dropped while vortexing. Following an overnight fixation at 4°C, the cells were pelleted by centrifugation and incubated with 50 µL (100 U) of RNAse (Qiagen, Valencia, CA) at room temperature for 20 min. Propidium iodide (500 µL of 10 µg/mL) (Sigma) was added to each cell suspension and the cells were kept in the dark at 4°C overnight.

Cells were then analyzed by flow cytometry on a FACSCalibur instrument (BD Biosciences, San Jose, CA) using the doublet discrimination module, and data were acquired using CellQuest (BD Biosciences) software. The cell cycle was modeled using ModFit software (Venty Software, Topsham, ME).

## Supplementary Material

Suppl

## Figures and Tables

**Table 1:** Antiproliferative activity of β-Carbolines on human colon and pancreatic cancer cell lines.

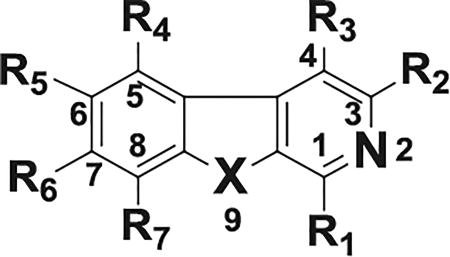												
Compound No.	R_1_	R_2_	R_3_	R_4_	R_5_	R_6_	R_7_	X	HCT116 (CC^a^) IC_50_ (µM)	HPAC (PC^b^) IC_50_ (µM)	Mia- PaCa2 (PC^b^) IC_50_ (µM)	Panc-1 (PC^b^) IC_50_ (µM)
**1**	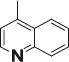	H	H	H	H	OCH_3_	H	NH	7.0	ND^c^	ND	ND
**2**	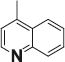	H	H	H	OCH_3_	H	H	NH	0.51	0.56	0.49	0.58
**3**	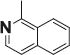	H	H	H	H	OCH_3_	H	NH	ND	34.77	49.17	>50.00
**4**	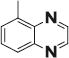	H	H	H	OCH_3_	H	H	NH	26.10	>50.00	>50.00	>50.00
**5**	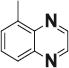	H	H	H	H	OCH_3_	H	NH	49.60	>50.00	>50.00	>50.00
**6**	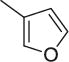	H	H	H	OCH_3_	H	H	NH	>50.00	45.11	38.28	>50.00
**7**	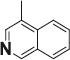	H	H	H	OCH_3_	H	H	NH	5.70	7.37	4.70	10.46
**8**	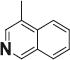	H	H	H	H	OCH_3_	H	NH	33.50	44.88	36.73	>50.00
**9**	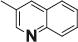	H	H	H	OCH_3_	H	H	NH	14.10	21.88	17.02	28.96
**10**	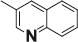	H	H	H	H	OCH_3_	H	NH	13.10	14.45	16.72	37.21
**11**	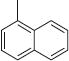	H	H	H	OCH_3_	H	H	NH	0.13	0.29	0.20	0.29
**12**	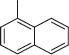	H	H	H	H	OCH_3_	H	NH	3.60	25.54	6.61	>50.00
**13**	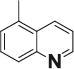	H	H	H	OCH_3_	H	H	NH	0.90	5.83	2.58	7.52
**14**	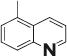	H	H	H	H	OCH_3_	H	NH	12.80	>50.00	28.55	>50.00
**15**	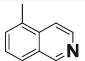	H	H	H	OCH_3_	H	H	NH	9.00	15.92	9.81	21.20
**16**	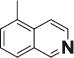	H	H	H	H	OCH_3_	H	NH	15.10	34.40	24.06	>50.00
**17**	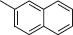	H	H	H	OCH_3_	H	H	NH	0.67	5.57	1.36	8.40
**18**	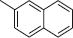	H	H	H	H	OCH_3_	H	NH	17.10	20.76	21.68	48.03
**19**	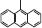	H	H	H	OCH_3_	H	H	NH	2.10	6.08	2.43	10.49
**20**	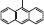	H	H	H	H	OCH_3_	H	NH	1.70	5.47	3.59	14.94
**21**	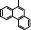	H	H	H	OCH_3_	H	H	NH	0.53	0.54	0.84	17.97
**22**	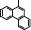	H	H	H	H	OCH_3_	H	NH	0.87	5.67	1.57	>50.00
**23**	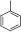	H	H	H	OCH_3_	H	H	NH	33.50	44.79	37.05	>50.00
**24**	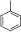	H	H	H	H	OCH_3_	H	NH	31.40	34.27	34.30	>50.00
**25**	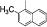	H	H	H	OCH_3_	H	H	NH	1.80	9.38	2.23	19.76
**26**	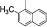	H	H	H	H	OCH_3_	H	NH	2.43	17.63	20.74	33.28
**27**	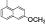	H	H	H	OCH_3_	H	H	NH	2.22	23.12	47.19	>50.00
**28**	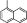	H	H	H	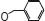	H	H	NH	ND	0.83	0.51	0.57
**29**	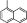	H	H	H	H	H	CH3	NH	2.60	6.39	3.54	>50.00
**30**	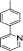	H	H	H	OCH_3_	H	H	NH	3.00	17.03	48.40	47.35
**31**	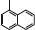	H	H	H	H	H	OCH_3_	NH	ND	20.54	31.90	26.49
**32**	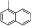	H	H	H	OH	H	H	NH	ND	ND	ND	ND
**33**	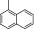	H	Ph	H	OCH_3_	H	H	NH	ND	7.72	3.63	>10
**34**	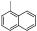	H	H	H	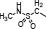	H	H	NH	ND	3.83	0.58	6.11
**35**	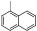	H	H	CH3	H	H	H	NH	ND	4.03	1.17	4.56
**36**	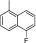	H	H	H	OCH_3_	H	H	NH	ND	2.37	0.33	2.56
**37**	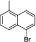	H	H	H	OCH_3_	H	H	NH	ND	6.18	1.17	2.92
**38**	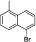	H	H	H	CH3	H	H	NH	ND	>50.00	41.67	>50.00
**39**	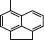	H	H	H	OCH_3_	H	H	NH	ND	4.79	2.95	>50.00
**40**	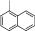	H	H	H	Cl	H	H	S	ND	49.97	13.50	>50.00
**41**	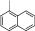	H	H	H	H	H	H	O	ND	>50.00	33.59	>50.00
**42**	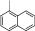	H	H	H	H	H	H	S	ND	>50	>50	>50
**43**	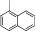	H	H	H	H	H	H	NH	ND	2.470	1.70	9.83
**44**	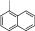	H	H	H	CH3	H	H	NH	ND	>50	13.08	>50
**45**	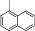	H	H	H	OCF3	H	H	NH	ND	35.48	8.63	>50
**46**	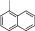	H	H	H	CN	H	H	NH	ND	>50.00	40.41	>50
**47**	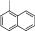	CO2Et	H	H	OCH_3_	H	H	NH	ND	12.86	17.52	23.28
**48**	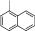	H	H	H	H	H	H	NCH3	ND	21.23	37.35	33.86
**49**	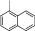	H	H	H	OCH_3_	H	H	NCH3	ND	44.19	34.02	41.27

a CC = Colon cancer;

b PC = Pancreatic cancer;

c ND = Not determined

**Table 2:** Antiproliferative activity of selected β-carbolines on human breast cancer cell lines.

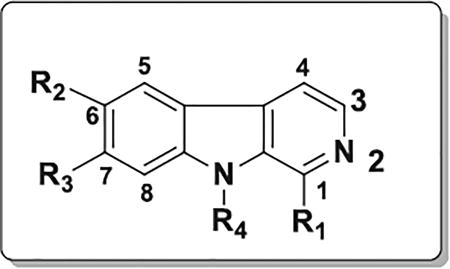						
Compound Number	R_1_	R2	R_3_	R_4_	MCF-7 IC_50_ (µM)	MDA-MB-468 IC50 (µM)
**11**	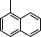	OCH_3_	H	H	0.26	0.08
**34**	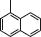	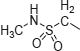	H	H	2.04	1.11
**43**	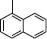	H	H	H	1.42	0.80
**45**	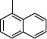	OCF_3_	H	H	7.61	4.99
**48**	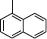	H	H	CH_3_	50.80	14.23

**Table 3:** Antiproliferative activity of selected β-carbolines against human metastatic melanoma cancer cell lines.

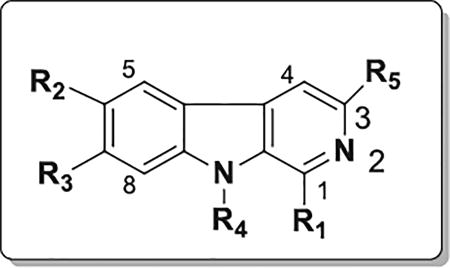							
Compound Number	R_1_	R_2_	R_3_	R_4_	R_5_	A375 IC_50_ (µM)	WM164 IC_50_ (µM)
**2**	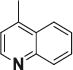	OCH_3_	H	H	H	0.92 ± 0.10	0.38 ± 0.41
**11**	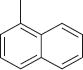	OCH_3_	H	H	H	0.23 ± 0.07	0.13 ± 0.02
**36**	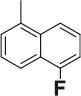	OCH_3_	H	H	H	0.76 ± 0.10	0.56 ± 0.13
**37**	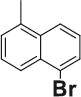	OCH_3_	H	H	H	3.97 ± 0.46	2.18 ± 0.16
**39**	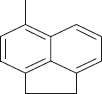	OCH_3_	H	H	H	3.51 ± 0.18	2.54 ± 0.10
**47**	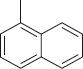	OCH_3_	H	H	CO_2_Et	13.91 ± 0.88	6.61 ± 0.85
**48**	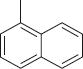	H	H	CH_3_	H	4.16 ± 343	2.09 ± 0.08

**Table 4:** Ligand-Receptor Interaction Energies.

Molecule	Total Ligand- Receptor Interaction Energy	Detailed Interaction Energy terms	
Compound **11**	−10.67 Kcal/mol	Ligand-Receptor Van der Waals Energy	−8.50 Kcal/mol
		Ligand-Receptor Electrostatic Energy	0.03 Kcal/mol
		Ligand-Receptor Hydrogen Bond Energy	−0.69 Kcal/mol
		Ligand-Receptor Solvation Free Energy	−1.61 Kcal/mol
		Ligand-Receptor Conformational Entropy	0.09 Kcal/mol

Compound **12**	−9.86 Kcal/mol	Ligand-Receptor Van der Waals Energy	−8.49 Kcal/mol
		Ligand-Receptor Electrostatic Energy	0.08 Kcal/mol
		Ligand-Receptor Hydrogen Bond Energy	−0.32 Kcal/mol
		Ligand-Receptor Solvation Free Energy	−1.23 Kcal/mol
		Ligand-Receptor Conformational Entropy	0.09 Kcal/mol

Compound **2**	−10.48 Kcal/mol	Ligand-Receptor Van der Waals Energy	−8.84 Kcal/mol
		Ligand-Receptor Electrostatic Energy	0.03 Kcal/mol
		Ligand-Receptor Hydrogen Bond Energy	−1.01 Kcal/mol
		Ligand-Receptor Solvation Free Energy	−0.76 Kcal/mol
		Ligand-Receptor Conformational Entropy	0.09 Kcal/mol

Compound **49**	−8.82 Kcal/mol	Ligand-Receptor Van der Waals Energy	−7.61 Kcal/mol
		Ligand-Receptor Electrostatic Energy	−0.01 Kcal/mol
		Ligand-Receptor Hydrogen Bond Energy	−0.66 Kcal/mol
		Ligand-Receptor Solvation Free Energy	−0.63 Kcal/mol
		Ligand-Receptor Conformational Entropy	0.09 Kcal/mol
