# Supplementary material for: Synthesis, Biological Evaluation and Modeling Studies of New Pyrido[3,4-b]indole Derivatives as Broad-Spectrum Potent Anticancer Agents
Source: Drug Des. Author manuscript; Available in PMC 2018 Mar 1. (PMC5771418; doi:10.4172/2169-0138.1000143)
Supplement: Suppl [file NIHMS871086-supplement-Suppl.pdf]

Synthesis, Biological Evaluation and Modeling Studies of New Pyrido[3,4-*b*]indole Derivatives as Potent Broad-Spectrum Anticancer Agents

Shivaputra A. Patil[1,†], James K. Addo[1,#], Hemantkumar Deokar[2], Shan Sun[1,±] , Jin Wang[1], Wei Li[1], Parker D. Suttle[3], Wei Wang[4], Ruiwen Zhang[4*], John K. Buolamwini[1,2,*]

Supplemental Material

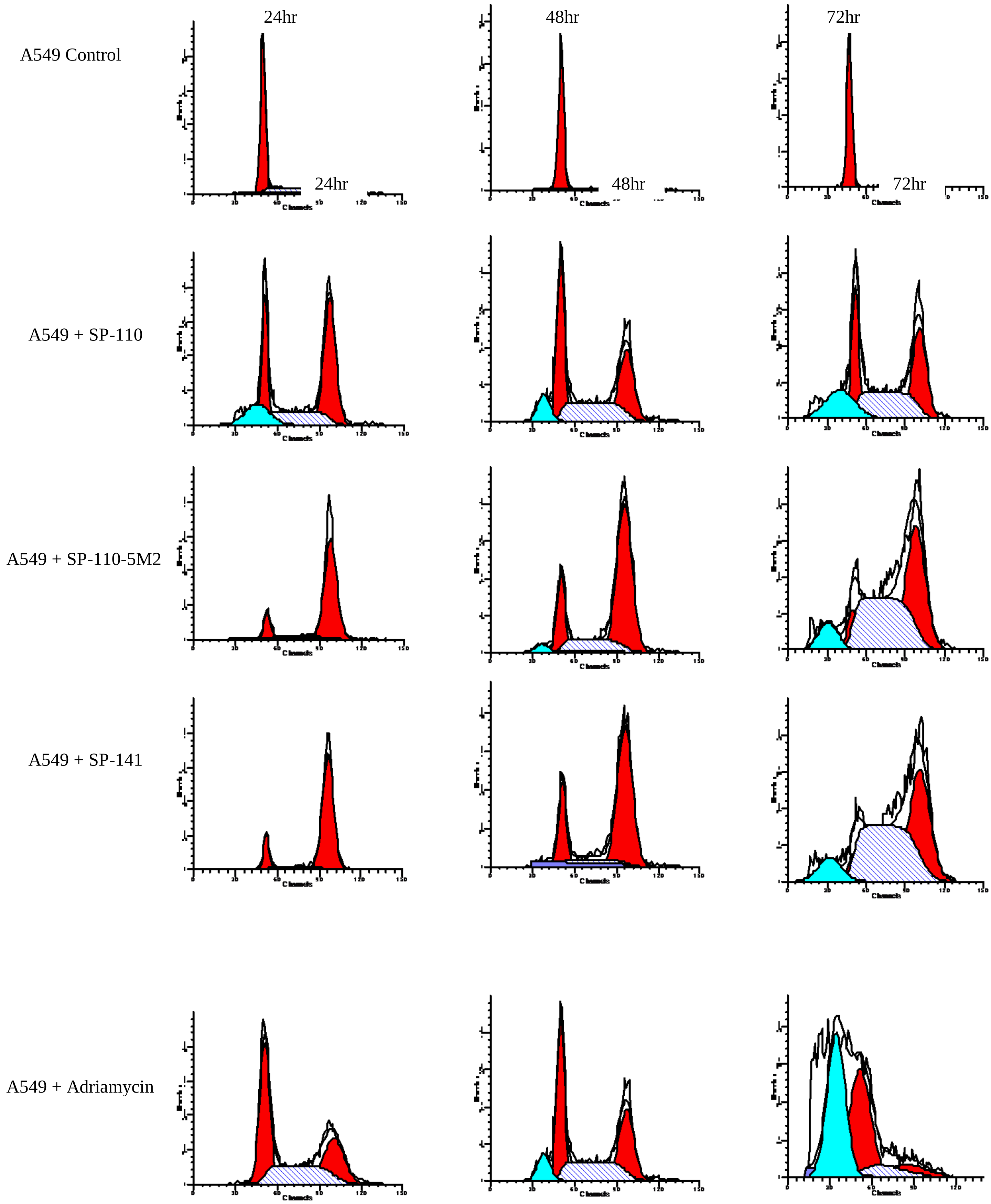
24hr
48hr
72hr
A549 Control
A549 + SP-110
A549 + SP-110-5M2
A549 + SP-141
A549 + Adriamycin

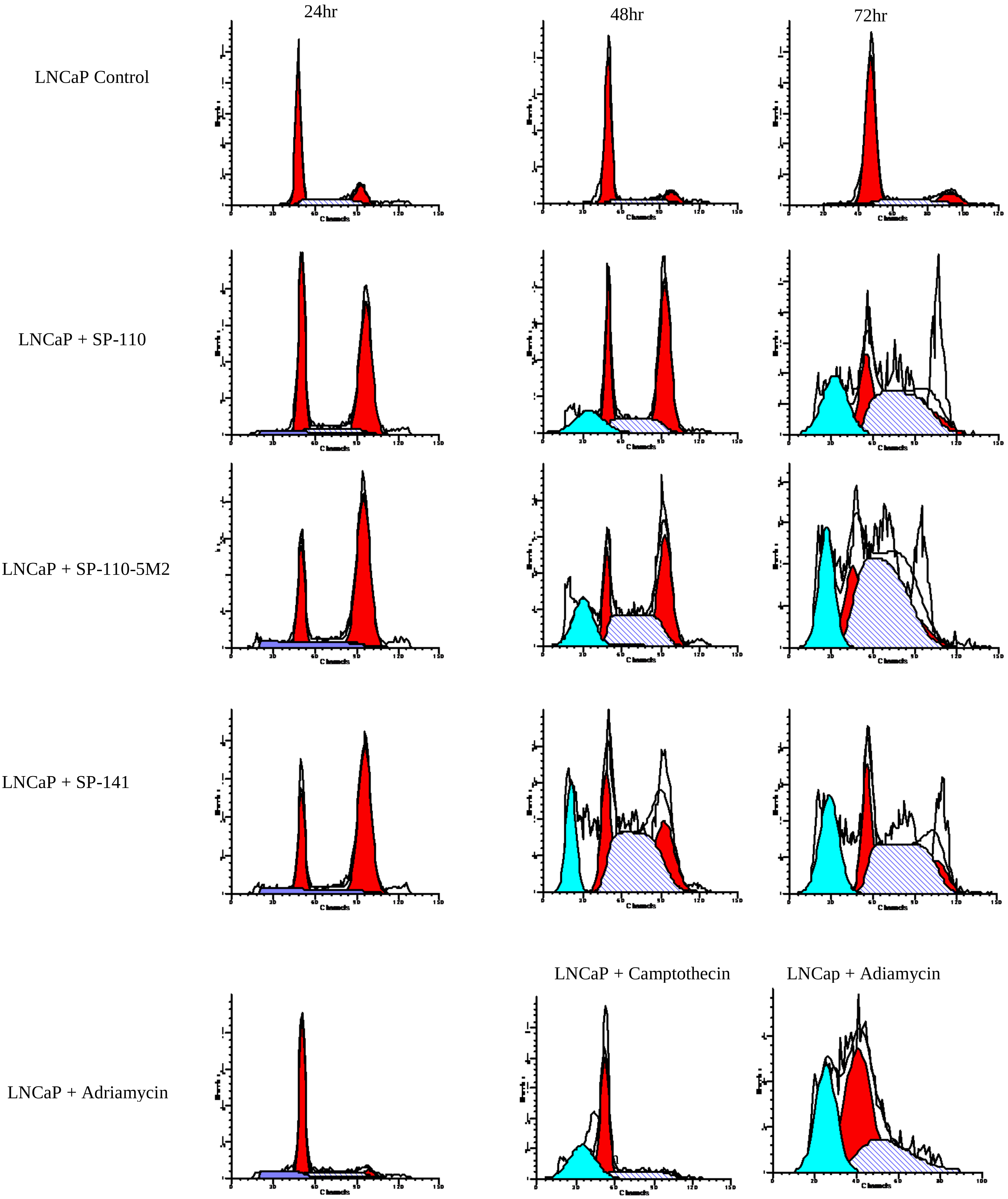
24hr
48hr
72hr
LNCaP Control
LNCaP + SP-110
LNCaP + SP-110-5M2
LNCaP + SP-141
LNCaP + Camptothecin
LNCap + Adiamycin
LNCaP + Adriamycin

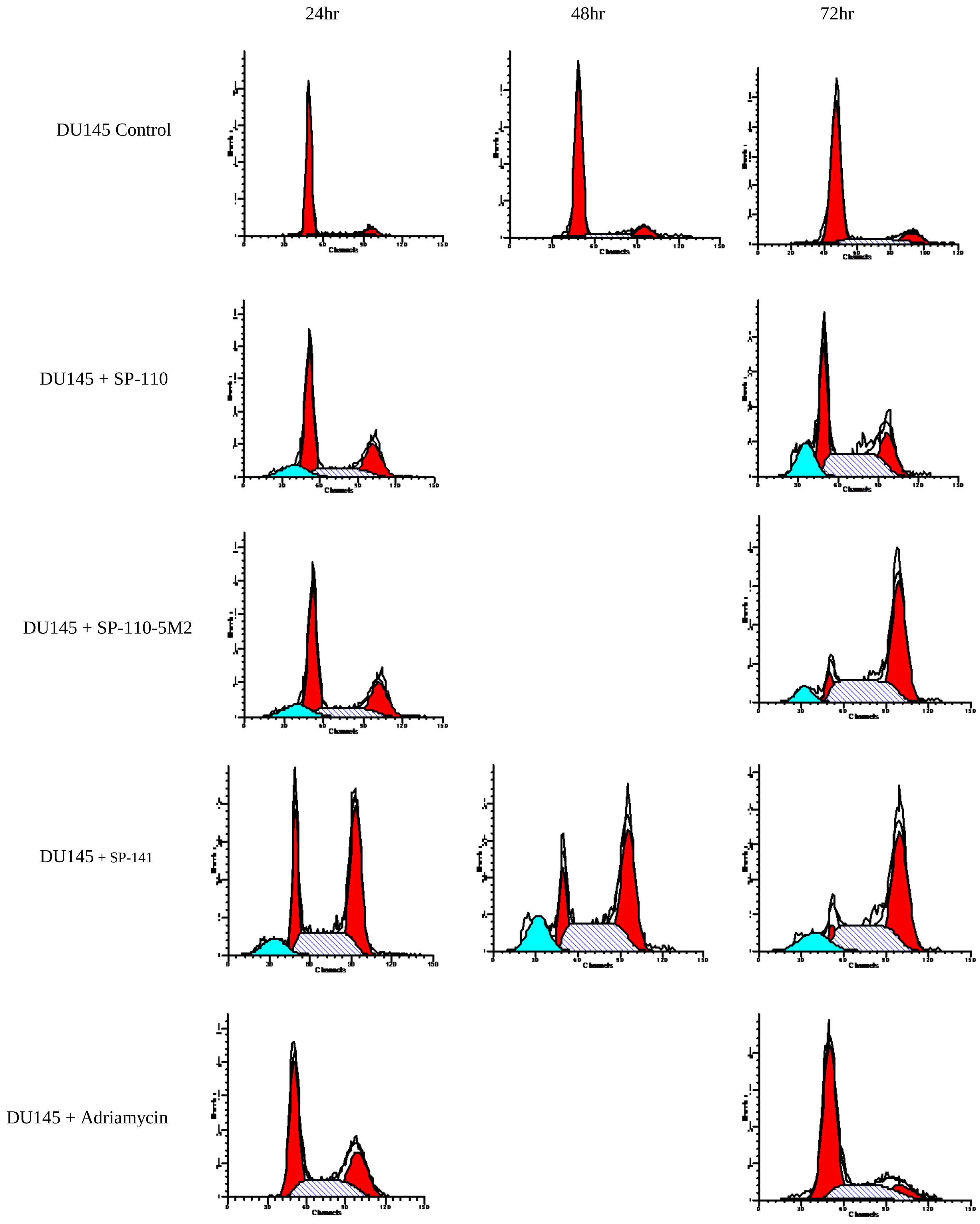
24hr
48hr
72hr
DU145 Control
DU145 + SP-110
DU145 + SP-110-5M2
DU145 + SP-141
DU145 + Adriamycin
Channels

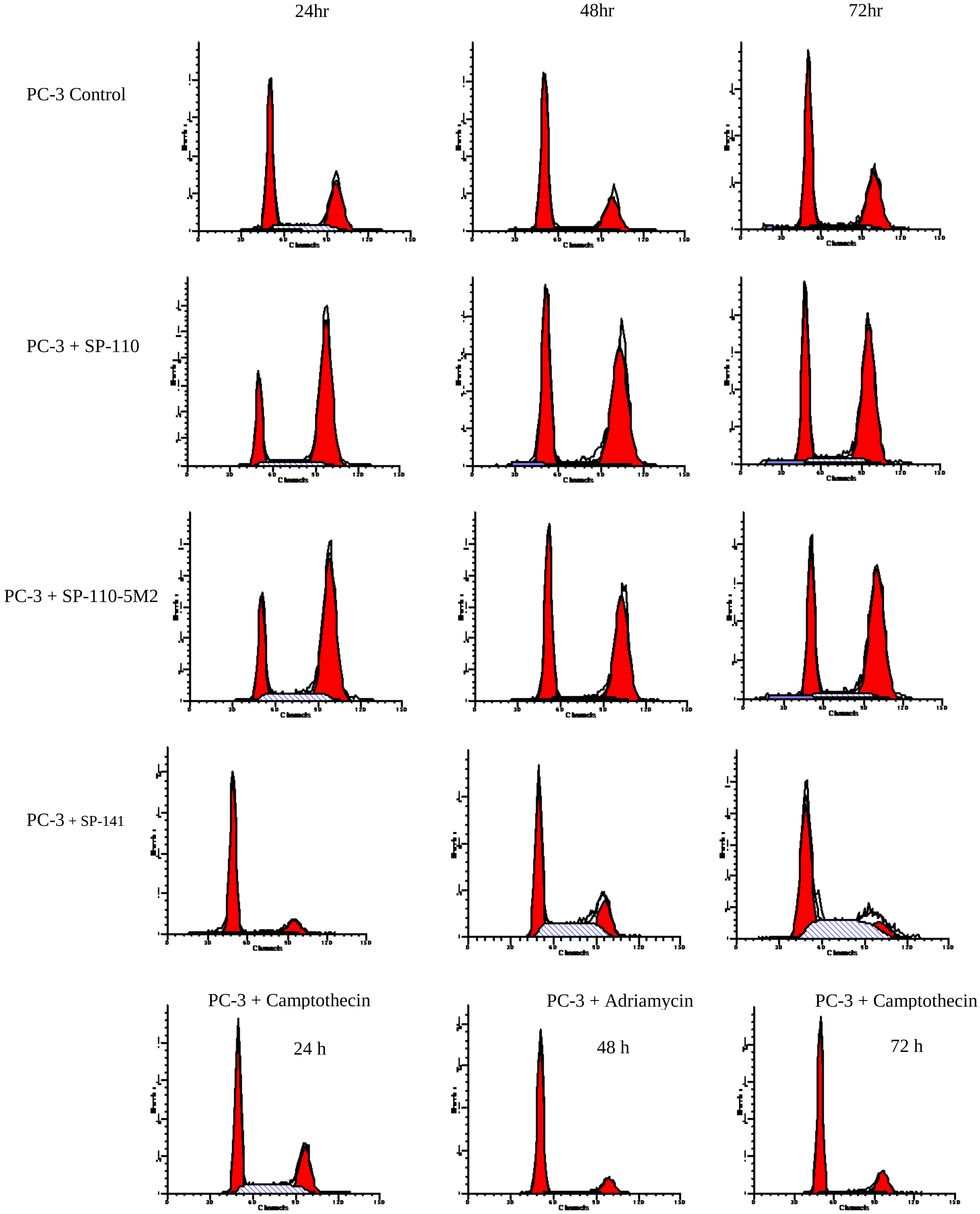
24hr
48hr
72hr
PC-3 Control
PC-3 + SP-110
PC-3 + SP-110-5M2
PC-3 + SP-141
PC-3 + Camptothecin
24 h
PC-3 + Adriamycin
48 h
PC-3 + Camptothecin
72 h
Channels

	24hr	48hr	72hr
HCT116 WT Control			
HCT116 WT + SP-110			
HCT116 WT + SP-5M2			
HCT116 WT + SP-141			
HCT116 WT + Adriamycin			

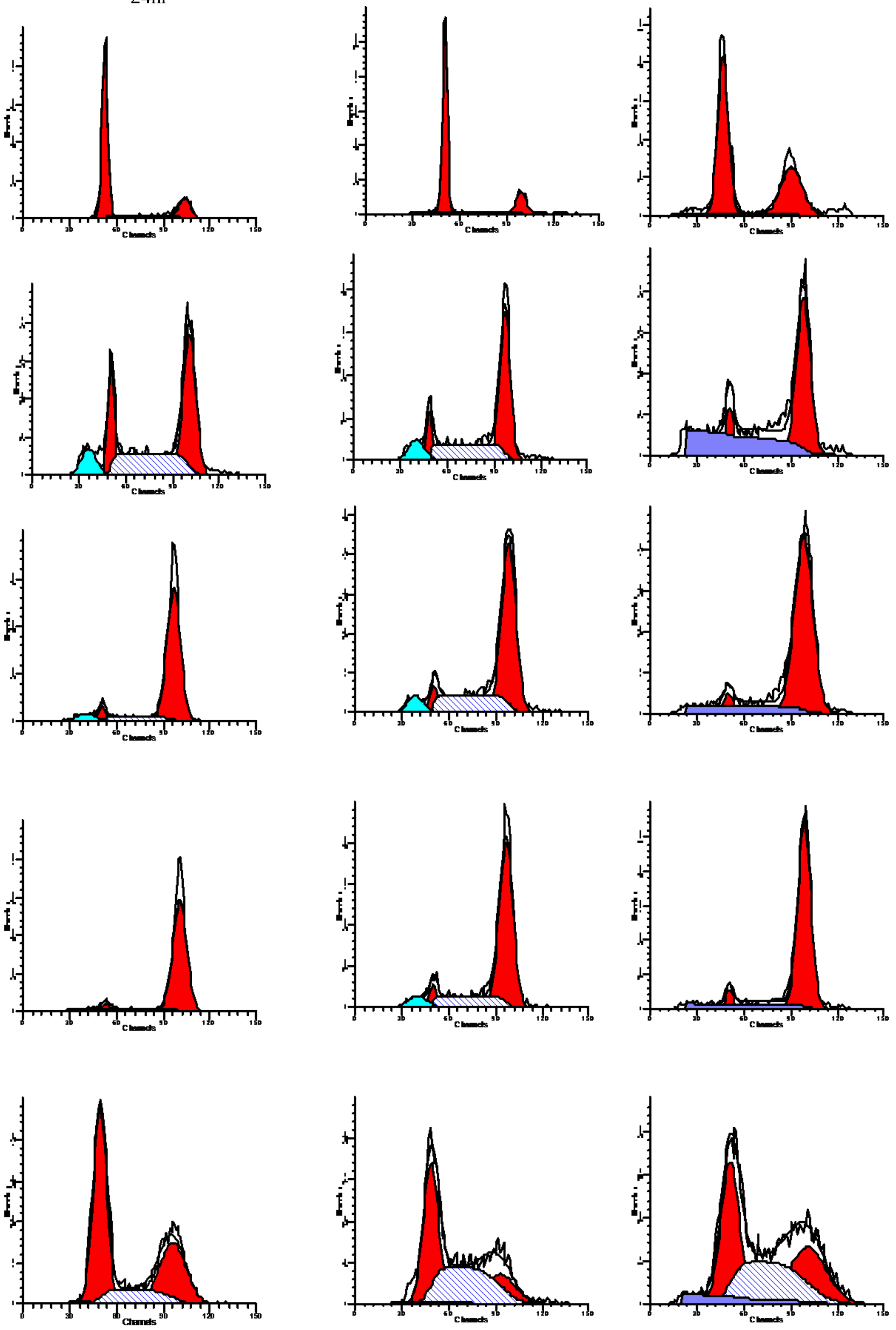

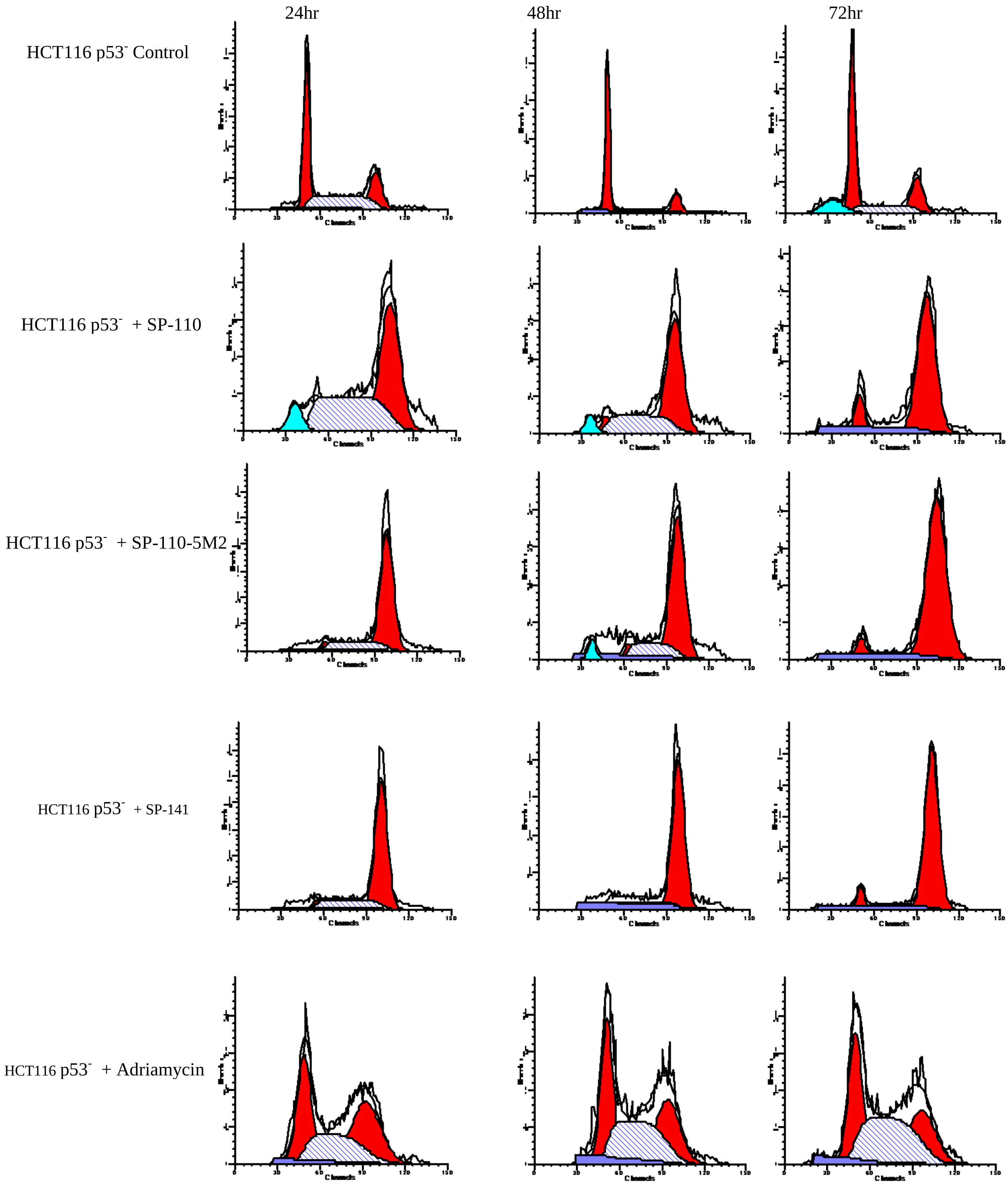

24hr
48hr
72hr
HCT116 p53- Control
HCT116 p53- + SP-110
HCT116 p53- + SP-110-5M2
HCT116 p53- + SP-141
HCT116 p53- + Adriamycin

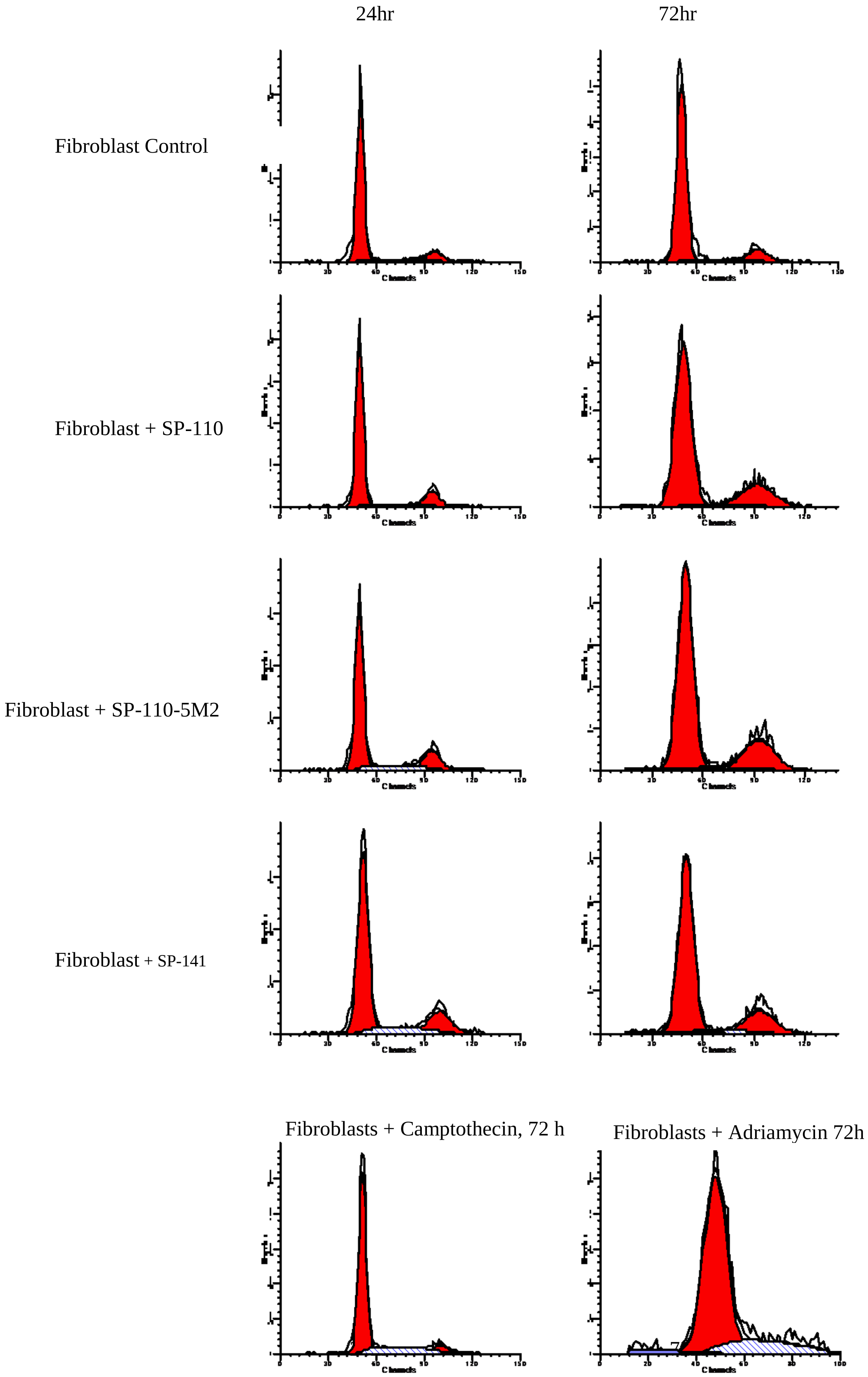
24hr
72hr
Fibroblast Control
Fibroblast + SP-110
Fibroblast + SP-110-5M2
Fibroblast + SP-141
Fibroblasts + Camptothecin, 72 h
Fibroblasts + Adriamycin 72h

Figure S1. The effects of various compounds on the cell cycles of different cancer cell lines and normal human fibroblast cell line. Cells were treated with or without 10 μM compound. G0/G1 and G2/M phases cell populations are represented by left and right red peaks, respectively. The S phase population is in between the G0/G1 and the G2/M phases. The sub-G0/G1 cyan peak indicates the apoptotic cell population. Blue regions represent debris.
